# Eco-Friendly Synthesis, Biological Evaluation, and *In Silico* Molecular Docking Approach of Some New Quinoline Derivatives as Potential Antioxidant and Antibacterial Agents

**DOI:** 10.3389/fchem.2021.679967

**Published:** 2021-06-10

**Authors:** Ahmed M. El-Saghier, Mohamed El-Naggar, Abdel Haleem M. Hussein, Abu-Bakr A. El-Adasy, M. Olish, Aboubakr H. Abdelmonsef

**Affiliations:** ^1^ Chemistry Department, Faculty of Science, Sohag University, Sohag, Egypt; ^2^ Chemistry Department, Faculty of Sciences, University of Sharjah, Sharjah, United Arab Emirates; ^3^ Chemistry Department, Faculty of Science, Al-Azhar University, Assiut, Egypt; ^4^ Chemistry Department, Faculty of Science, South Valley University, Qena, Egypt

**Keywords:** multicomponent reaction, chromenoquinolines, antioxidant, antibacterial, molecular docking

## Abstract

A new series of quinoline derivatives **5–12** were efficiently synthesized *via* one-pot multicomponent reaction (MCR) of resorcinol, aromatic aldehydes, β-ketoesters, and aliphatic/aromatic amines under solvent-free conditions. All products were obtained in excellent yields, pure at low-cost processing, and short time. The structures of all compounds were characterized by means of spectral and elemental analyses. In addition, all the synthesized compounds **5–12** were *in vitro* screened for their antioxidant and antibacterial activity. Moreover, *in silico* molecular docking studies of the new quinoline derivatives with the target enzymes, human NAD (P)H dehydrogenase (quinone 1) and DNA gyrase, were achieved to endorse their binding affinities and to understand ligand–enzyme possible intermolecular interactions. Compound **9** displayed promising antioxidant and antibacterial activity, as well as it was found to have the highest negative binding energy of -9.1 and -9.3 kcal/mol for human NAD (P)H dehydrogenase (quinone 1) and DNA gyrase, respectively. Further, it complied with the Lipinski’s rule of five, Veber, and Ghose. Therefore, the quinoline analogue **9** could be promising chemical scaffold for the development of future drug candidates as antioxidant and antibacterial agents.

## Introduction

Quinolines are very important compounds used for new drug development. They are reported as highly selective cytotoxic (Luo et al., 2009; Bawa et al., 2010; Meshram et al., 2012; Sidoryk et al., 2015), broad-spectrum antimicrobial (including activity against *Mycobacterium tuberculosis* as well as HIV-1 integrase inhibition activity) (Wang et al., 2019), antileishmanial (Asif, 2014), anticonvulsant, anti-inflammatory, cardiovascular activity (Acharyulu et al., 2008; Kumar, Bawa, and Gupta, 2010), and have antidiabetic effect (Shang, et al., 2018a).

Moreover, natural and synthetic chromene moiety attached to quinolines has important biological activity such as anticancer (El-Maghraby, 2014), anticoagulant, antispasmolytic (El-Maghraby and Aboubakr, 2019), antiangiogenesis (Sangani et al., 2012), antimicrobial (Gómez and Vladimir, 2013), anti-inflammatory (Asif, 2014), anti-invasive (Sidoryk et al., 2015), antioxidant (Shang, et al., 2018b), analgesic, and anticonvulsant agents (Dua et al., 2011). Therefore, many researchers have synthesized these compounds as target structures and were evaluated for their biological activity.

Recently, there is a growing demand for the development of organic reactions in eco-friendly media. Synthetic manipulations have to be made to minimize the use of hazardous chemicals by replacing the traditional organic solvents in reactions and their subsequent workup with other nontoxic and environmentally benign solvents such as water.

For complementing this way and in continuation of our search work (Abdelmonsef and Mosallam, 2020; Haredi Abdelmonsef et al., 2020; Noser et al., 2020; Rashdan et al., 2020; Shehadi et al., 2020; Gomha et al., 2021), we decided to eco-friendly synthesize some quinoline scaffolds and test *in vitro* their antioxidant and antibacterial activity. Moreover, the compounds were docked into the binding sites of the target enzymes: human NAD (P)H dehydrogenase (quinone 1) and DNA gyrase, respectively. In addition, adsorption, distribution, metabolic, excretion, and toxicity (ADME/T) properties of the newly synthesized compounds were also calculated.

## Materials and Methods

### Chemistry

All melting points were measured by a Stuart SMP10 Melting point apparatus. IR spectra (KBr) were recorded by using a Bruker spectrometer (ν, cm^−1^). ^1^H-NMR, ^13^C-NMR, and DEPT 135 spectra were recorded on 400 and 100 MHz, DMSO-d_6_ at AVANCE-III 400 MHz High performance FT-NMR Spectrum BRUKER. Bio Spin International AG-Switzerland at Sohag University. Elemental analysis was carried out at the Microanalytical Research Center, Faculty of Science, Cairo University. All the chemicals were commercially available from Sigma-Aldrich and El-Gomhouria Company, Egypt.

#### General Procedure for Synthesis of Compounds 5–12

A mixture of resorcinol **1** (1.10 g, 10 mmol), different aromatic aldehydes, namely, benzaldehyde (1.06 g, 10 mmol), 4-chlorobenzaldehyde (1.40 g, 10 mmol), 2-chlorobenzaldehyde (1.40 g, 10 mmol), 4-methylbenzaldehyde (1.20 g, 10 mmol) **2a-d**, 2-hydroxybenzaldehyde (1.22 g, 10 mmol) **2e**, and/or different β-ketoesters, namely, ethyl cyanoacetate (1.13 g, 10 mmol) 3a, ethyl acetoacetate (1.30 g, 10 mmol) 3b, and/or different aromatic or aliphatic amines, namely, 4-aminophenol (1.09 g, 10 mmol) 4b, benzyl amine (1.07 g, 10 mmol) 4c, was refluxed in an oil bath at 110°C for one hour. After completion of the transformation, the reaction mixture was cooled to RT, and then water was added (50 ml). The product was collected by filtration, then washed with water repeatedly, and recrystallized from appropriate solvents (25 ml) to give compounds **5a-d, 6, 7, 9, 10, 11,** and **12.**

##### Ethyl 2-amino-7-hydroxy-4-phenyl-1,4-dihydroquinoline-3-carboxylate (5a)

**Yield** (90%), **color:** brown crystals, **mp** = 115–117°C, IR (KBr) ν cm^-1^; 3450 (OH), 3325 (NH), 3213, 3201 (NH_2_), and 1739 (C=O). ^**1**^ **H-NMR** (400 MHz, DMSO-d_6_): δ 1.08 (t, 3H, CH_3_), 4.18 (q, 2H, CH_2_), 4.65 (s, 1H, CH-pyridine), 6.22–7.28 (m, 10H, Ar-H+NH_2_), 8.46 (s, 1H, NH), and 9.05 (s, 1H, OH). ^**13**^ **C-NMR** (100 MHz, DMSO-d_6_): δ 14.32, 60.00, 103.72, 112.28, 120.98, 128.24, 129.20, 129.68, 129.95, 131.48, 132.19, 141.32, 152.48, 155.86, 158.28, 167.94, and 173.64. **Anal. Calcd.** for **C**_**18**_ **H**_**18**_ **N**_**2**_ **O**_**3**_ (310.35): C, 69.66; H, 5.85; N, 9.03%. **Found** C, 70.00; H, 5.34; N, 9.20%.

##### Ethyl 2-amino-4-(4-chlorophenyl)-7-hydroxy-1,4-dihydro-quinoline-3-carboxylate (5b)

**Yield** (97%), **color**: orange crystals, **mp** = 138–140°C; **IR (KBr) ν** cm^-1^; 3442 (OH), 3332 (NH), 3246, 3231 (NH_2_), and 1738 (C=O). ^**1**^ **H-NMR** (400 MHz, DMSO-d_6_): δ 1.02 (t, 3H, CH_3_), 4.40 (q, 2H, CH_2_), 5.81 (s, 1H, CH-pyridine), 6.29–7.37 (m, 10H, Ar-H+NH_2_), 8.88 (s, 1H, NH), and 9.01 (s, 1H, OH). ^**13**^ **C-NMR** (100 MHz, DMSO-d_6_): δ 14.42, 60.00, 103.92, 112.28, 120.98, 128.24, 129.21, 129.68, 129.95, 131.48, 132.19, 141.32, 152.48, 155.86, 158.28, 167.94, and 173.64. **Anal. Calcd.** for **C**_**18**_ **H**_**17**_ **ClN**_**2**_ **O**_**3**_ (344.79): C, 62.70; H, 4.57; Cl, 10.28; N, 8.12%. **Found C**, 62.50; H, 4.73; Cl, 10.37; N, 8.27%.

##### Ethyl 2-amino-4-(2-chlorophenyl)-7-hydroxy-1,4-dihydro-quinoline-3-carboxylate (5c)

**Yield** (95%), color: reddish crystals, **mp** = 140–142°C, IR (KBr) ν cm^-1^; 3420 (OH), 3364 (NH), 3192, 3175 (NH_2_), and 1741 (C=O). ^**1**^ **H-NMR** (400 MHz, DMSO-d_6_): δ H = 1.21 (t, 3H, CH_3_), 4.74 (q, 2H, CH_2_), 5.03 (s, 1H, CH-pyridine), 6.60–7.36 (m, 9H, Ar-H+NH_2_), 8.22 (s, 1H, NH), and 9.19 (s, 1H, OH). ^**13**^ **C-NMR** (100 MHz, DMSO-d_6_): δ 14.35, 56.30, 103.14, 106.32, 114.90, 119.57, 127.27, 128.79, 129.52, 130.52, 133.14, 139.09, 152.92, 156.04, 158.50, 167.67, and 173.16. **Anal. Calcd.** for **C**_**18**_ **H**_**17**_ **ClN**_**2**_ **O**_**3**_ **(344.79)**: C, 62.70; H, 4.57; Cl, 10.28; N, 8.12%. **Found C**, 62.56; H, 4.39; Cl, 10.30; N, 8.06%.

##### Ethyl 2-amino-7-hydroxy-4-(p-tolyl)-1,4-dihydroquinoline-3-carboxylate (5d)

**Yield** (93%), color: reddish crystals, mp = 107–109°C, IR (KBr) ν cm^-1^; 3351, 3329, 3294, 3260 (OH, NH, NH_2_), and 1739 (C=O). ^**1**^ **H-NMR** (400 MHz, DMSO-d_6_): δ 1.20 (t, 3H, CH_3_), 2.32 (s, 3H, CH_3_), 4.08 (q, 2H, CH_2_), 5.71 (s, 1H, CH-pyridine), 6.27–7.28 (m, 9H, Ar-H+NH_2_), 7.58 (s, 1H, NH), and 8.77 (s, 1H, OH). ^**13**^ **C-NMR** (100 MHz, DMSO-d_6_): δ 21.01, 21.49, 59.96, 102.89, 112.15, 125.77, 128.66, 128.99, 129.36, 129.63, 137.81, 139.81, 155.82, and 168.18. Dept. 135 **NMR** (100 MHZ, DMSO-d6): δ (+) 21.02, 21.49, (-) 37.24, (+) 102.88, 105.85, 112.15, 115.77, 128.66, 128.99, 129.36, 129.79, and 139.92. **Anal. Calcd.** for **C**_**19**_ **H**_**20**_ **N**_**2**_ **O**_**3**_ (324.37): C, 70.35; H, 6.21; N, 8.64%. **Found** C, 70.68; H, 5.66; N, 8.57%.

##### 6-Amino-10-hydroxy-8,12b-dihydro-7H-chromeno[3,4-c]quinolin-7-one (6)

**Yield** (89%), color: yellow crystals, mp = 270–272°C, IR (KBr) ν cm^-1^; 3345, 3305, 3293, 3251 (OH, NH, NH_2_), and 1668 (C=O). ^**1**^ **H-NMR** (400 MHz, DMSO-d_6_): δ 2.38 (s, 1H, *sp* ^*3*^ CH), 5.95 (s, 1H, NH_2_), 6.21–7.66 (m, 8H, Ar-H+ CH-pyridine), 7.27 (s, 1H, NH), 9.98 (s, 1H, NH), and 11.12 (s, 1H, OH). ^**13**^ **C-NMR** (100 MHZ, DMSO-d_6_): δ 40.95, 44.62, 79.19, 97.70, 115.01, 117.67, 124.09, 125.22, 129.03, 131.54, 135.61, 135.82, 141.10, 141.32, 155.30, and 171.91. **Anal. Calcd.** for **C**_**16**_ **H**_**12**_ **N**_**2**_ **O**_**3**_ (280.28): C, 68.56; H, 4.32; N, 9.99%. **Found C**, 68.45; H, 4.67; N, 9.89%.

##### 10-Hydroxy-7-methyl-8,12b-dihydrodibenzo[c,f][2,7]na-phthayridin-6(5H)-one (7)

**Yield** (95%), **color:** yellow crystals, mp = 185–187°C, IR (KBr) ν cm^-1^; 3317, 3212 (OH, NH), and 1662 (C=O-amide). ^**1**^ **H-NMR** (400 MHz, DMSO-d_6_): δ H = 1.87 (s, 3H, CH_3_), 4.87 (s, 1H, *sp* ^*3*^CH CH-pyrane), 6.22–6.94 (m, 8H, Ar-H), 7.73 (s, 1H, NH), and 8.64 (s, 1H, OH). ^**13**^ **C-NMR** (100 MHz, DMSO-d_6_): δ 33.68, 42.60, 103.04, 103.41, 106.68, 116.12, 118.88, 119.32, 126.71, 128.55, 128.95, 130.09, 130.43, 153.60, 155.88, 156.12, and 158.96. **Anal. Calcd.** for **C**_**17**_ **H**_**14**_ **N**_**2**_ **O**_**2**_ (278.31): C, 73.37; H, 5.07; N, 10.0 7%. **Found C**, 73.41; H, 5.20; N, 10.00%.

##### 10-Hydroxy-8-(4-hydroxyphenyl)-6-methyl-8,12b-dihydro-7H-chromeno[3,4-c]quinolin-7-one (9)

**Yield** (92%), **color:** pale brown crystals, **mp** = 160–162°C, IR (KBr) ν cm^-1^; 3244 (2OH). ^**1**^ **H-NMR** (400 MHz, DMSO-d_6_): δ 1.94 (s, 3H, CH_3_), 5.27 (s, 1H, CH-pyridine), 6.76–7.33 (m, 12H, Ar-H), 8.90 (s, 1H, NH), and 11.53 (s, 1H, OH). ^**13**^ **C-NMR** (100 MHz, DMSO-d_6_): δ 19.23, 36.24, 102.94, 106.51, 108.85, 116.19, 116.44, 119.94, 121.26, 123.08, 127.19, 127.71, 128.32, 140.60, 142.92, 152.45, 155.15, 156.12, 157.44, and 160.64. **MS** (relative intensity) *m/z*: 371 (M, 5.1%), 289 (23%), 165 (35%), 105 (70%), and 44 (100%). **Anal. Calcd.** for **C**_**23**_ **H**_**17**_ **NO**_**4**_ (371.39): C, 74.38; H, 4.61; N, 3.77%. **Found C,** 74.76; H, 4.90; N, 3.70%.

##### 3-Acetyl-4-(4-chlorophenyl)-7-hydroxy-1-(4-hydroxy-phenyl)quinolin-2(1H)-one (10)

**Yield (**81%), **color:** dark red crystals, **mp** = 115–117°C, **IR (KBr) ν** cm^-1^; 3335, 3273 (2OH), and 1710 (C=O). ^**1**^ **H-NMR** (400 MHz, DMSO-d_6_): δ 1.95 (s, 3H, CH_3_), 6.22–6.94 (m, 11H, Ar-H), 8.42 (s, 1H, NH), and 11.56 (s, 2H, 2OH). ^**13**^ **C-NMR** (100 MHz, DMSO-d_6_): δ 19.75, 103.30, 115.78, 115.94, 116.06, 116.23, 123.05, 127.37, 128.82, 129.13, 129.30, 130.27, 135.83, 141.06, 142.74, 148.80, 150.82, 156.15, 157.02, and 172.53. **Anal. Calcd.** for **C**_**23**_ **H**_**16**_ **ClNO**_**4**_ (405.83): C, 68.07; H, 3.97; Cl, 8.74; N, 3.45%. **Found C,** 68.21; H, 3.39; Cl, 8.62; N, 3.47%.

##### Ethyl 2-amino-1-benzyl-7-hydroxy-4-phenyl-1,4-dihydro-quinoline-3-carboxylate (11)

**Yield** (89%), **color:** pink crystals, **mp** = 122–124°C, IR (KBr) ν cm^-1^; 3400, 3286, 3273 (OH, NH_2_), and 1700 (C=O). ^**1**^ **H-NMR** (400 MHz, DMSO-d_6_): δ 1.19 (t, 3H, CH_3_), 3.93 (q, 2H, CH_2_), 4.32 (s, 2H, CH_2_), 5.00 (s, 1H, CH-pyridine), 6.28–7.40 (m, 13H, Ar-H+NH_2_), and 8.93 (s, 1H, OH**)**. ^**13**^ **C-NMR** (100 MHz, DMSO-d_6_): δ 14.13, 49.67, 53.68, 60.73, 100.10, 115.01, 127.67, 127.97, 128.59, 128.60, 128.63, 128.80, 128.70, 128.85, 128.92, 128.95, 129.03, 129.23, 135.61, 136.74, 141.50, 141.65, 154.44, 156.64, and 165.12. **Anal. Calcd.** for **C**_**25**_ **H**_**24**_ **N**_**2**_ **O**_**3**_ (400.47): C, 74.98; H, 6.04; N, 7.00%. **Found C**, 74.99; H, 6.01; N, 7.20%.

##### 10-Hydroxy-8-(4-hydroxyphenyl)-6-imino-6H-chromeno[3,4-c]quinolin-7(8H)-one (12)

**Yield** (97%), **color:** dark red crystals, **mp** = 260–262°C, **IR (KBr) ν** cm^-1^; 3432, 3238, 3227 (OH, NH_2_), and 1708 (C=O). ^**1**^ **H-NMR** (400 MHz, DMSO-d_6_): δ 4.31 (s, 1H, CH-pyridine), 6.76–7.43 (m, 12H, Ar-H+NH_2_), and 9.24 (s, 2H, 2OH). ^**13**^ **C-NMR** (100 MHz, DMSO-d_6_): δ 49.67, 100.10, 113.72, 115.01, 116.46, 116.66, 122.72, 123.65, 123.85, 126.77, 129.03, 129.13, 129.23, 131.33, 135.61, 141.65, 144.73, 154.12, 154.44, 155.80, 156.64, and 165.12. **Anal. Calcd.** for **C**_**22**_ **H**_**14**_ **N**_**2**_ **O**_**4**_ (370.36): C, 71.35; H, 3.81; N, 7.56%. **Found C**, 71.22; H, 3.48; N, 7.52%.

### Biological Study

#### *In-Vitro* Antioxidant Assay

The total antioxidant capacity of the compounds was evaluated according to the method described by Prieto et al. (1999). An aliquot of 0.5 ml of sample solution was combined with 4.5 ml of reagent solution (0.6 M sulfuric acid, 28 mM sodium phosphate, and 4 mM ammonium molybdate). In case of blank, 0.5 ml of 45% DMSO (dimethyl sulphoxide) has been used. The tubes incubated in a boiling water bath at 95°C for 90 min. After the samples cooled at RT, the absorbance of the aqueous solution of each sample was measured at 695 nm against blank by using a UV-2450 spectrophotometer (Shimadzu, Japan). The total antioxidant activity was expressed as the absorbance of the sample at 695 nm. The higher absorbance value indicated higher antioxidant activity (Wan et al., 2011).

#### Antimicrobial Assay

The antimicrobial activity of compounds was tested *in vitro* against various bacterial strains, Gram-negative (*Escherichia coli*), and Gram-positive (*Staphylococcus haemolyticus, Kocuria kristinae, Enterococcus casseliflavus,* and *Bacillus subtilis*) identified in Al-Azhar University, Regional Center for Mycology and Biotechnology. Amikacin, levofloxacin, and gentamicin were used as standard antibacterial agents obtained from Bioanalyse® Ltd. (Turkey) for the comparison of biological activity of newly synthesized molecules.

The method applied is “modified agar diffusion” (Bauer et al., 1966) using 2.0 mg per disc used to determine the antimicrobial activity. Nutrient agar (ready for use from EDM company,Egypt) inoculated with microbial cell suspension into sterile Petri dishes (200 µl in 20 ml medium). Sterile paper discs of 6 mm diameter saturated with a tested compound placed on the surface of the inoculated agar plates and negative control done using paper discs loaded with 20 µl of DMSO. Incubate overnight (24 h) at 37°C. Inhibition zone was measured at the end of the incubation period.

### *In Silico* Docking Protocol

The molecular docking study of all compounds was carried out to identify their plausible mode of action against the active site residues of the target enzymes.

The 2D structures of the newly synthetic compounds were accurately drawn using ChemDraw Ultra 7.0 software and then converted to SDF format using Open Babel GUI tool (O’Boyle et al., 2011). An in-house library of ten synthesized compounds was generated for further study. The enzymes of NQO1: human NAD (P)H dehydrogenase (quinone 1) (PDB code 1DXO) (Faig et al., 2000) and DNA gyrase (PDB code 1JA6) (Holdgate et al., 1997) were selected as targets for docking simulation. The crystal structures of the targets were retrieved from the RCSB Protein Data Bank web server. The protein files were optimized by removing the ligands and water molecules. The grid box was generated around the active site pocket. Subsequently, the docking process was achieved using PyRx, virtual screening tool of AutoDock 4 software (Dallakyan and Olson, 2015). Among the nine confirmations of these ligand molecules obtained from the docking simulation, the pose with the lowest binding energy was selected for further study (HA and SP, 2016; Hussein et al., 2018). In addition, 7,8-Dihydroxyflavone (tropoflavin) and novobiocin were selected as standard drugs to compare the docking score with that of the synthesized compounds. Discovery Studio 3.5 was then used to visualize the intermolecular interactions between the ligand molecules and enzymes.

The adsorption, distribution, metabolic, excretion, and toxicity (ADME/T) analyses and physicochemical properties of the newly synthesized compounds were also calculated using admetSAR (http://lmmd.ecust.edu.cn/admetsar2) and Molinspiration (https://molinspiration. com/) free Web-based tools.

## Results and Discussion

### Chemistry

The present study entails the synthesis of a novel series of quinoline analogues 5**–12** *via* one-pot multicomponent reaction (MCR) under solvent-free conditions.

**SCHEME 1:**
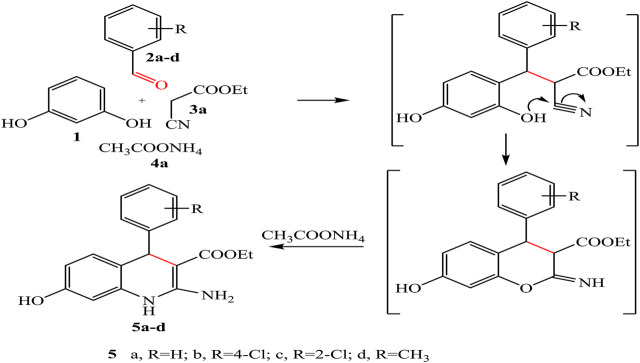
Synthesis of quinoline derivative 5a–d under solvent-free conditions.

Our synthesis begins with heating a mixture of resorcinol **1**, aromatic aldehyde, namely, benzaldehyde, 2-chlorobenzaldehyde, 4-chlorobenzaldehyde, 4-methyl benzaldehyde **2a-d,** and ethyl cyanoacetate **3a** in the presence of ammonium acetate **4a** to obtain quinoline derivatives **5a-d** under solvent-free conditions (Scheme 1).

The chemical structures of the products **5a-d** were established on the basis of IR, ^1^H-NMR, ^13^C-NMR spectral data, and elemental analysis.

For example, IR spectrum of compound **5d** showed new absorption bands at 3,351, 3,329, 3,294, 3,260 cm^-1^ for OH, NH, and NH_2_, respectively. The ^1^H-NMR spectrum of compound **5d** revealed triplet signal at 1.20 ppm for –CH_2_-CH_3_, singlet signal at 2.32 ppm for -CH_3_ attached to phenyl ring, quartet signal at 4.08 ppm for -CH_2_, singlet signal at 5.71 ppm for CH-pyridine, singlet signal at 7.58 ppm for NH group, and singlet signal at 8.77 ppm for OH group, beside the appearance of the amino protons in interference with the aromatic protons at 6.27–7.28 ppm as a singlet. In addition, ^13^C-NMR spectrum of compound **5d** showed signals at 21.02, 21.49, 59.96, and 168.18 ppm assigned to 2CH_3_ and CH_2_ and acetyl carbonyl group, respectively. Moreover, a negative signal of the CH_2_ group was obtained at 61.59 ppm in DEPT 135 spectrum.

**SCHEME 2:**
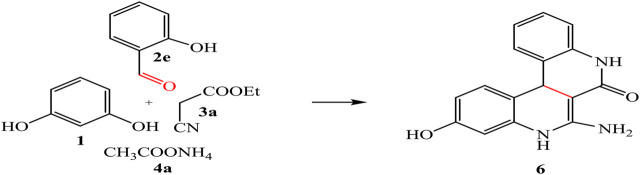
Synthesis of quinoline 6.

By analogy, multicomponent reaction of resorcinol **1**, aromatic aldehyde **2e**, ethyl cyanoacetate **3a**, and ammonium acetate **4a** afforded the quinoline derivative **6** (Scheme 2).

IR spectrum of compound **6** showed band at 1,668 cm^-1^ referred to carbonyl group; in addition, its ^1^H-NMR spectrum exhibited beside the aromatic signals; new singlet signals at 11.12, 9.98, 7.27, 5.95, and 2.38 ppm were consistent with the OH, 2NH, NH_2_, and *sp* ^*3*^CH groups, respectively.

**SCHEME 3:**
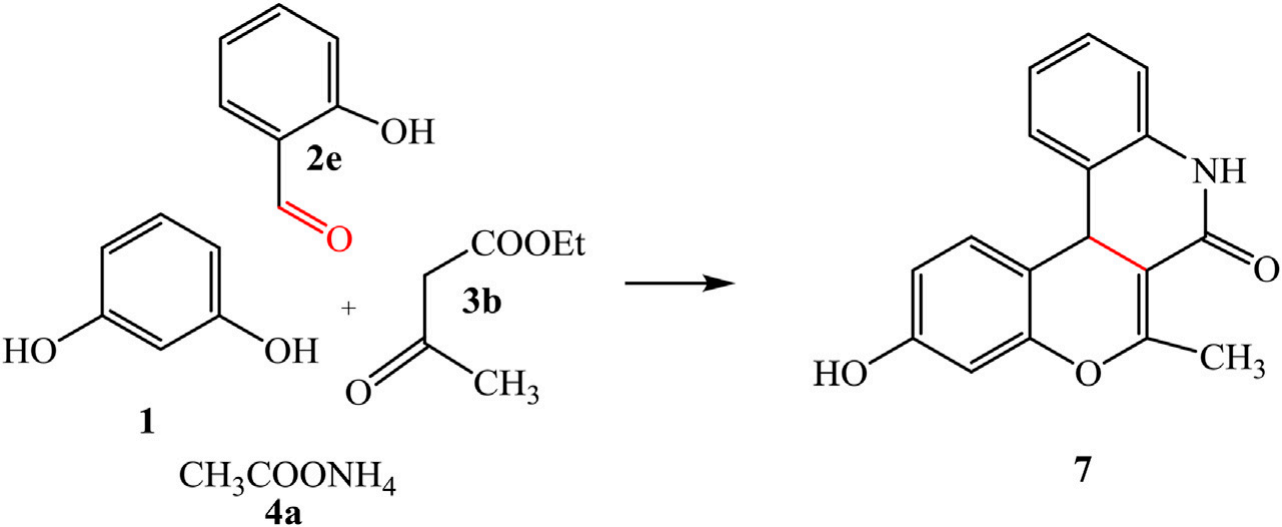
Schematic representation of the synthesis of component 7.

By adding ethyl acetoacetate **3b** instead of ethyl cyanoacetate **3a**, afforded the quinoline analogue **7** (Scheme 3). The chemical structure of compound **7** was established by elemental analysis and spectroscopic data, where IR spectrum showed amide carbonyl at 1,662 cm^-1^. ^1^H-NMR spectrum revealed a singlet signal at 1.87 ppm referred to CH_3_, 4.87 ppm for *sp* ^*3*^CH-pyrane, and OH at 8.64 ppm beside signals at 6.22–6.94 ppm for aromatic protons. Moreover, ^13^C-NMR spectrum represented a signal at 33.68 ppm referred to CH_3_, in addition to other signals which confirmed the chemical structure of 7.

**SCHEME 4:**
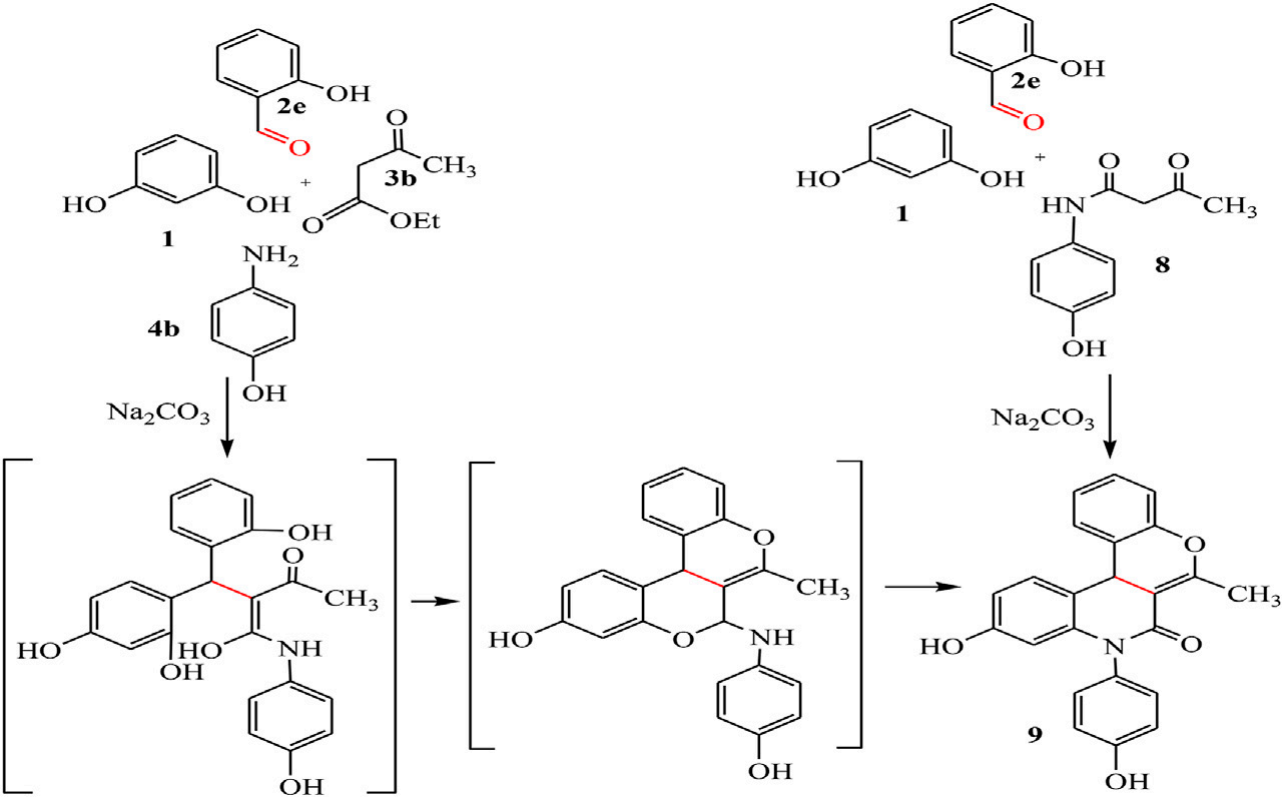
Synthetic pathway for compound 9.

By the same way, a multicomponent reaction of resorcinol **1** with aromatic aldehyde **2e**, ethyl acetoacetate **3b,** and *p*-aminophenol 4b in the presence of sodium carbonate under solvent-free conditions afforded the quinoline derivative **9**. This result was also achieved by the reaction of 3-oxobutanamide **8** with resorcinol **1** and salicylaldehyde **2e** in the presence of sodium carbonate which makes the medium alkaline, as declared in Scheme 4.

IR spectrum confirmed the chemical structure of compound **9** by disappearance of the characteristic bands for acetyl carbonyl and the presence of new bands referred to imide carbonyl at 1,635 cm^-1^. ^1^H-NMR spectrum showed a singlet signal for CH_3_ at 1.94 ppm, at 5.27 ppm a singlet signal referred to CH-pyridine, NH appeared at 8.90 ppm, and OH found at 11.53 ppm ^13^C-NMR spectrum revealed signals at 19.32 and 160.64 ppm referred to CH_3_ and imide carbonyl groups, respectively. Mass spectrum confirmed the molecular formula C_23_H_17_NO_4_ by the molecular ion peak at *m/z* 371.

**SCHEME 5:**
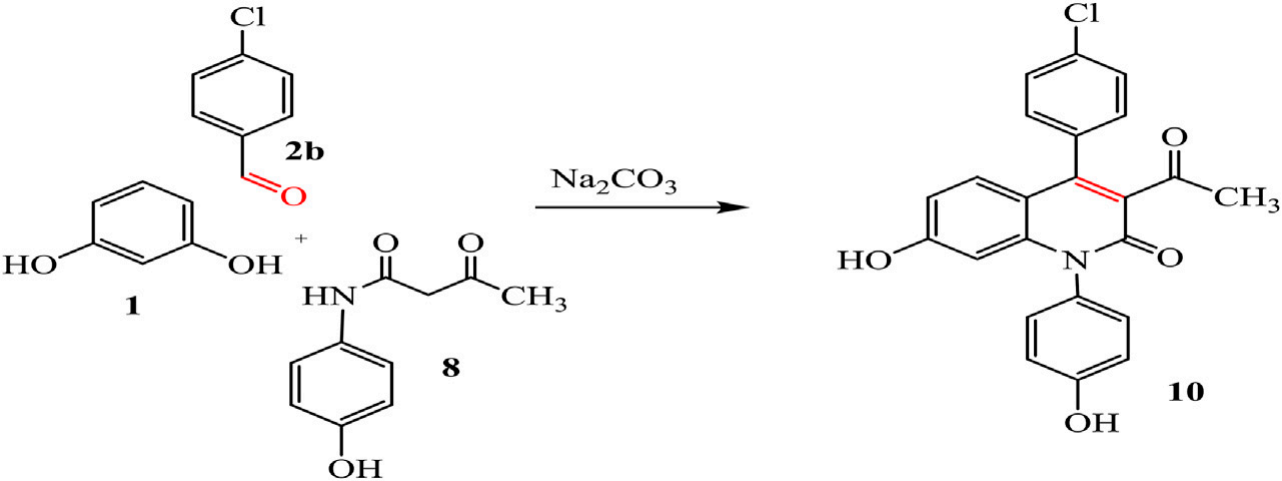
Synthesis of quinoline analogue 10.

By the same way, the treatment of resorcinol **1** with 4-chlorobenzaldehyde **2b** and 3-oxobutanamide **8** afforded the quinoline derivative **10**, as shown in Scheme 5.

IR spectrum of compound **10** showed new bands for acetyl and two OH groups at 1,710, 3,335, and 3,273 cm^-1^, respectively. Its ^1^H-NMR showed a singlet signal at 1.95 cm^-1^ for CH_3_, a singlet signal at 5.02 ppm for CH-pyridine, and two singlet signals at 8.42 and 11.56 ppm referred to two hydroxyl groups, beside multiplet signals of aromatic protons. Further, ^13^C-NMR spectrum confirmed the presence of methyl group at 19.75 ppm, imide carbonyl at 157 ppm, and acetyl carbonyl at 172.53 ppm.

**SCHEME 6:**
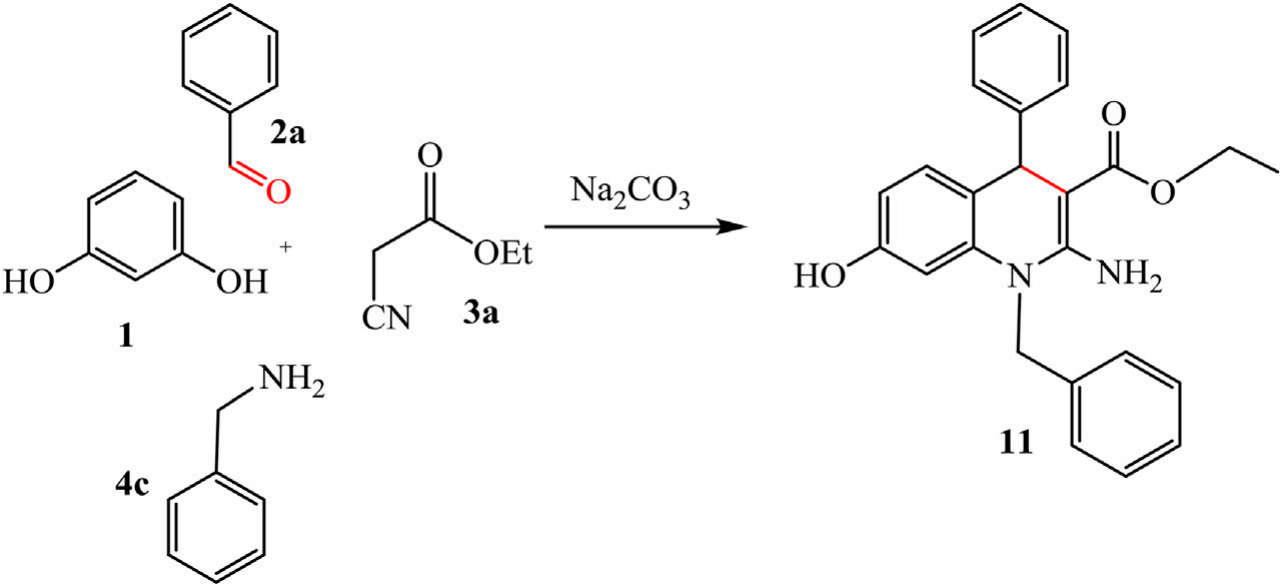
Synthesis of compound 11.

**SCHEME 7:**
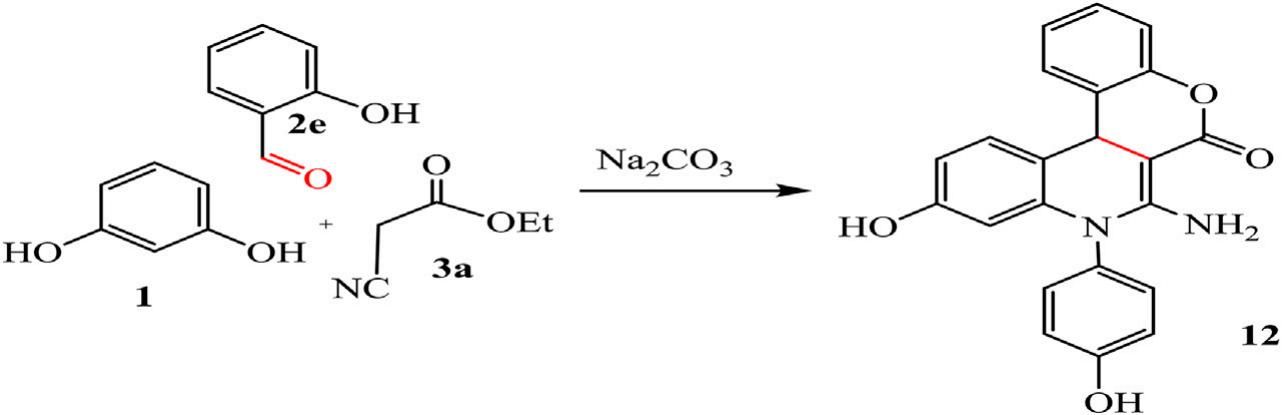
Synthesis of compound 12.

The reaction of resorcinol **1** with aldehyde, ethyl cyanoacetate, and aromatic amine was also studied to afford the quinoline analogues **11** and **12**. For compound **11** stopped at the step of cyclization but compound **12** formed by additional reaction with hydroxyl group on aldehyde with ester and H_2_O get out. Ester form disappeared from compound **12** while it revealed at compound **11**, as represented in Schemes 6, 7.

The spectral data of the newly synthesized compounds are represented as Supplementary Figures S1–S27 in supplementary information file.

### Biological Evaluation

#### *In-Vitro* Antioxidant Assay

The total antioxidant activity was determined using the phosphor molybdenum blue complex with a maximum absorption at 695 nm. The data presented in Table 1 showed that the tested compound **9** is the most active as represented in the following order: vit C > 9 > 12 > 5d > 11 > 5b.

**TABLE 1:** Antioxidant activity of compounds **5–12**.

Sample	Absorbance
Standard	1.03
5a	0.00
5b	0.14
5c	0.00
5d	0.26
6	0.00
7	0.00
9	0.38
10	0.00
11	0.22
12	0.29

#### Antimicrobial Evaluation

All the tested compounds showed good activity against bacterial strains tested with inhibition zones in range 9.0–20.0 mm. Amikacin showed inhibition zone 15.0 mm, levofloxacin 14.0–16.0 mm, and gentamicin 20.0 mm, as shown in Table 2. (Supplementary Figure S28 in supplementary information file). The compound **9** with electron donating groups like (-OH) and (-CH_3_), piperidine, and pyran moieties showed the strongest activity against Gram-negative (*Escherichia coli*) and Gram-positive (*Enterococcus casseliflavus*) strains.

**TABLE 2:** Antibacterial activity of the screened compounds **5–12**.

Inhibition zone (mm)	*Escherichia coli*	*Staphylococcus haemolyticus*	*Kocuria kristinae*	*Enterococcus casseliflavus*	*Bacillus subtilis*
Code no.					
Control	0	0	0	0	0
5a	12	9	8	10	9
5b	3	0	0	0	0
5c	0	0	0	0	0
5d	7	9	17	12	12
6	0	0	0	0	0
7	0	0	0	0	0
9	18	1	4	19	10
10	17	10	17	18	11
11	10	7	1	6	8
12	10	20	13	18	0
Standard	*Amikacin*	*Levofloxacin*	*Levofloxacin*	*Levofloxacin*	*Gentamicin*
St. Result	15	14	16	16	20

### Molecular Docking Protocol

To understand as well as to support the *in vitro* antioxidant and antibacterial activity of the newly synthesized compounds for the rational design of novel and potential inhibitor molecules, molecular docking studies were performed (Abdelmonsef et al., 2016; Dasari et al., 2017; Rondla et al., 2017; Abdelmonsef, 2019).

**FIGURE 1:**
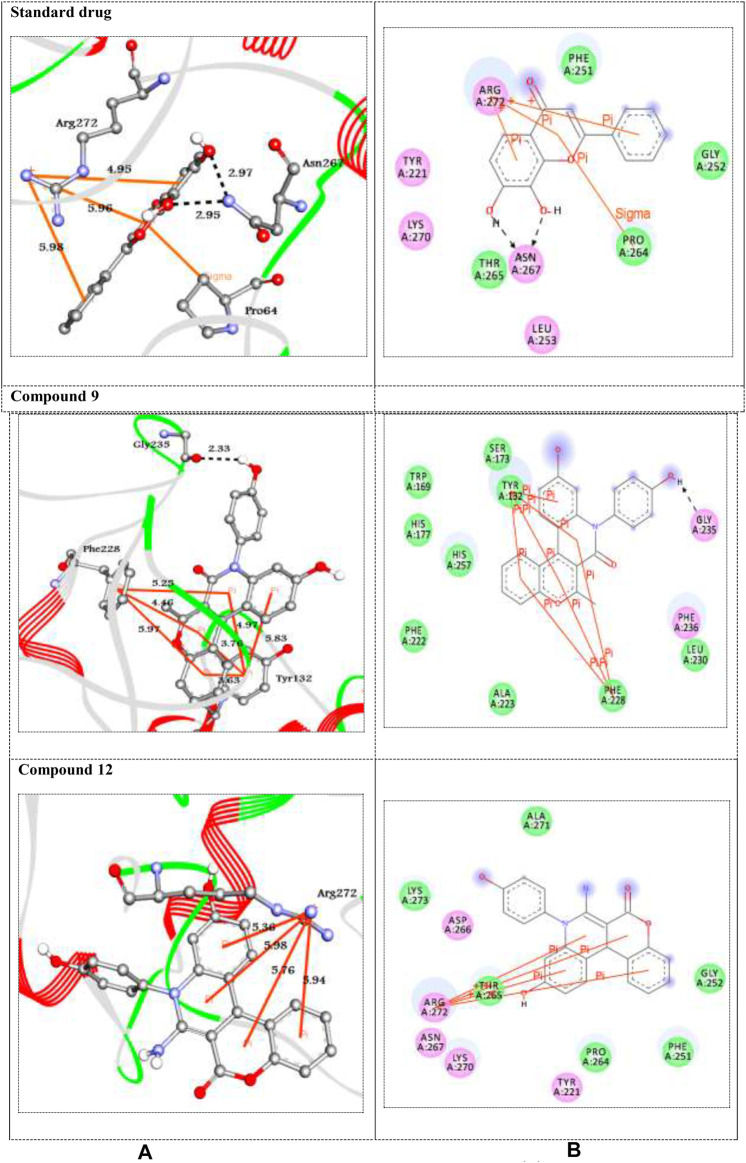
**(A)** 3D and **(B)** 2D representations of standard drug and the best docked compounds with 1DXO. H-bonds are represented in black dotted lines while pi-interactions are shown in orange lines. Colored balls with 3-letter code represented the amino acid residues of the target 1DXO.

**FIGURE 2:**
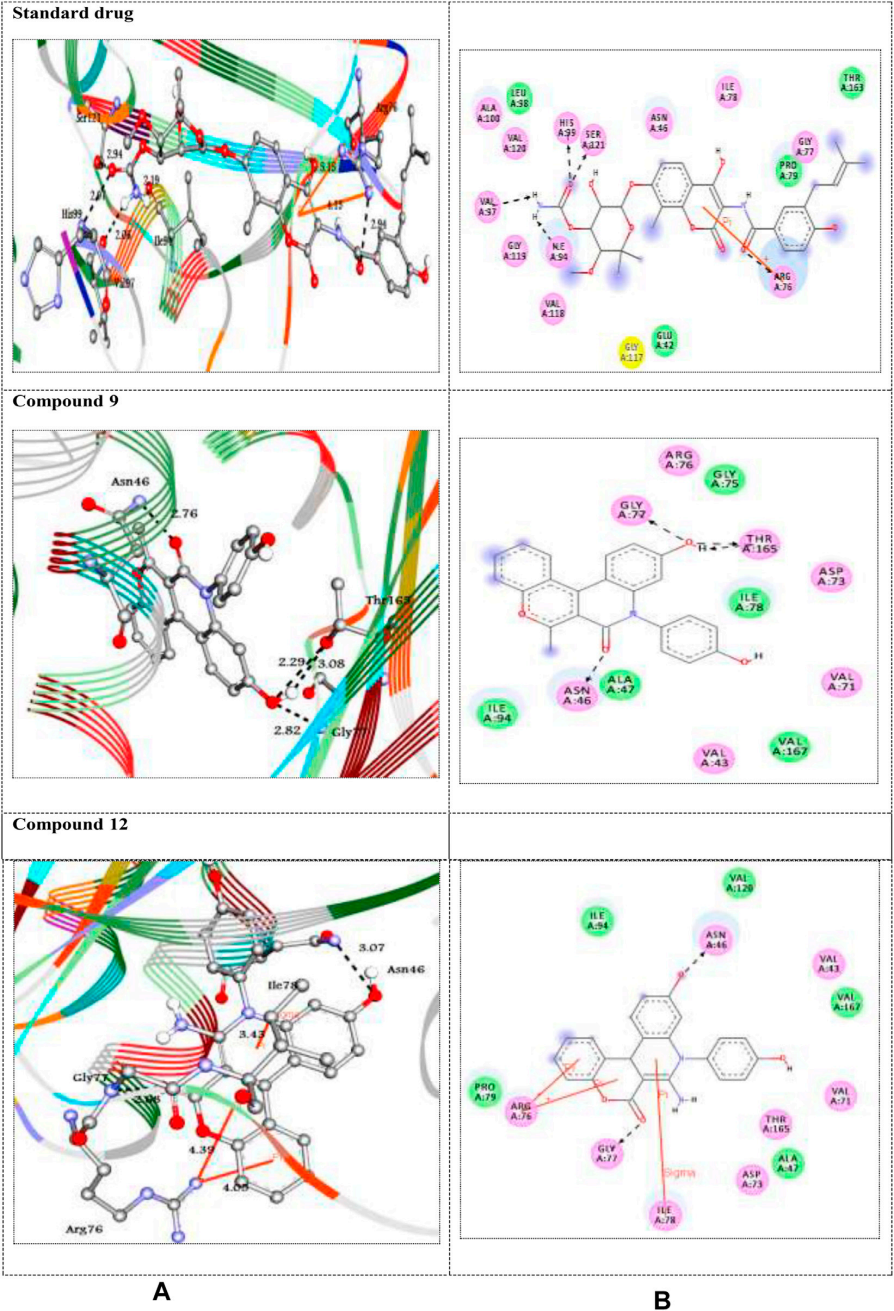
**(A)** 3D and **(B)** 2D representations of standard drug and the best docked compounds standard drug with 1JA6. H-bonds are represented in black dotted lines while pi-interactions are shown in orange lines. Colored balls with 3-letter code represented the amino acid residues of the target 1JA6.

Here, *in silico* molecular docking simulation of standard drugs and the new ten molecules with the active site of the target enzymes 1DXO and 1AJ6 was carried out to evaluate their binding affinities and to understand ligand–enzyme possible intermolecular interactions. The docking energies (ΔG_bind_) and amino acid interactions for the screened compounds were summarized in Table 3. The 2D and 3D representation of the best docked complexes were represented in Figures 1, 2.

**TABLE 3:** The binding energies (ΔG_bind_) of the docked standard drugs and compounds **5–12** and their intermolecular interactions with the active site of the target enzymes.

	Antioxidant			Antibacterial		
	(ΔG_bind_)	Docked complex (amino acid–ligand) interactions	Distance (Å)	(ΔG_bind_)	Docked complex (amino acid–ligand) interactions	Distance (Å)
Standard drug	−7.5	H-bonds		−8.3	H-bonds	
		Asn267:ND2―standard drug	2.97		Arg76:NH1― standard drug	2.94
		Asn267:ND2―standard drug	2.95		His99:N― standard drug	2.97
		π-cation interaction			Ser121:OG― standard drug	2.95
		Arg272:NH1―standard drug	4.95		Ile94:O― standard drug	2.19
		Arg272:NH1―standard drug	5.96		Val97:O― standard drug	2.06
		Arg272:NH1―standard drug	5.98		π-cation interaction	
		π-Sigma interaction			Arg76:NH1― standard drug	5.15
		Pro264:CB― standard. Drug	3.75		Arg76:NH2― standard drug	4.13
5a	−6.9	H-bonds		−7.7	H-bonds	
		Asn267:ND2―compound5a	3.09		Thr165:OG1―compound5a	3.09
		Asn267:O―compound5a	1.98		Thr165:OG1―compound5a	2.35
		π-cation interaction			π-cation interaction	
		Arg272:NH1―compound5a	5.98		Arg76:NH2―compound5a	4.28
		Arg272:NH2―compound5a	4.30			
5b	−7.0	H-bonds		−7.9	H-bonds	
		Asn267:ND2―compound5b	3.04		Thr165:OG1―compound5b	2.96
		Asn267:O―compound5b	1.86		Thr165:OG1―compound5b	2.47
		π-cation interaction			Asp73:OD1―compound5b	2.49
		Arg272:NH1―compound5b	5.97		π-cation interaction	
		Arg272:NH2―compound5b	4.61		Arg76:NH2―compound5b	3.96
5c	−7.1	H-bonds		−8.1	H-bonds	
		Asn267:ND2―compound5c	3.03		Thr165:OG1―compound5c	3.00
		Asn267:O―compound5c	2.18		Thr165:OG1―compound5c	2.15
		Lys270:O―compound5c	2.40		π-cation interaction	
		π-cation interaction			Arg76:NH1―compound5c	5.27
					Arg76:NH2―compound5c	3.84
		Arg272:NH2―compound5c	4.49			
5d	−7.7	H-bonds		−8.0	H-bonds	
		Tyr128:OH―compound5d	3.13		Thr165:OG1―compound5d	3.02
		π–π interaction			π-cation interaction	
		Tyr132―compound5d	5.00		Arg76:NH2―compound5d	3.89
		Phe236―compound5d	4.34			
		Phe228―compound5d	3.99			
6	−7.9	H-bonds		−8.6	H-bonds	
		Asn267:ND2―compound6	3.19		Gly77:N―compound6	3.08
		Lys270:O―compound6	2.22		Asp73:OD2―compound6	1.85
		π-cation interaction			Asp73:OD1―compound6	2.32
		Arg272:NH2―compound6	5.99			
7	−8.2	H-bonds		−7.8	H-bonds	
		Tyr126:OH―compound7	3.15		Thr165:OG1―compound7	3.11
		Tyr128:OH―compound7	3.14		π-cation interaction	
		π–π interaction			Arg76:NH2―compound7	3.91
		Phe178―compound7	5.64		Arg76:NH2―compound7	4.91
		Tyr126―compound7	4.38			
		Tyr126―compound7	4.38			
		Tyr126―compound7	4.76			
		Tyr126―compound7	5.12			
9	−9.1	H-bonds		−9.3	H-bonds	
		Gly235:O―compound9	2.33		Asn46:ND2―compound9	2.76
		π–π interaction			Gly77:N―compound9	2.82
		Tyr132―compound9	5.83		Thr165:OG1―compound9	3.08
		Tyr132―compound9	4.97		Thr165:OG1―compound9	2.29
		Tyr132―compound9	3.76			
		Tyr132―compound9	3.63			
		Phe228―compound9	5.25			
		Phe228―compound9	4.46			
		Phe228―compound9	5.97			
10	−8.0	H-bonds		−8.3	H-bonds	
		Lys270:O―compound10	2.14		Asn46:ND2―compound10	2.84
		π-cation interaction			Gly77:N―compound10	2.87
		Arg272:NH1―compound10	6.00		Thr165:OG1―compound10	2.92
		Arg272:NH2―compound10	3.95		π-cation interaction	
					Arg76:NH1―compound10	5.98
					Arg76:NH2―compound10	4.86
11	−7.7	H-bonds		−7.6	H-bonds	
		Asn267:ND2―compound11	3.20		Asn46:ND2―compound11	3.18
		Lys270:O―compound11	2.27		Gly77:N―compound11	2.65
		π-cation interaction			π-cation interaction	
		Arg272:NH2―compound11	3.46		Arg76:NH2―compound11	4.77
12	−8.7	π-cation interaction		−9.2	H-bonds	
		Arg272:NH1―compound12	5.36		Asn46:ND2―compound12	3.07
		Arg272:NH1―compound12	5.98		Gly77:N―compound12	2.68
		Arg272:NH1―compound12	5.76		π-cation interaction	
		Arg272:NH1―compound12	5.94		Arg76:NH2―compound12	4.39
					Arg76:NH2―compound12	4.05
					π-Sigma interaction	
					Ile78:CG1―compound12	3.43

#### Antioxidant Activity

Human NAD (P)H dehydrogenase (quinone 1) is an enzyme that combats the oxidative stress conditions (Dinkova-Kostova and Talalay, 2010; Atia and Abdullah, 2020) as a gene highly expressed in human adipocytes and performing its antioxidant activity (Palming et al., 2007). In the present study, the docking studies were performed against the crystal structure of human NAD (P)H dehydrogenase (quinone 1) with PDB code 1DXO. All the docked compounds were fit on the enzyme active site with the docking scores (ΔG_bind_) of the range -9.1–-6.9 kcal/mol; in addition, the standard drug exhibited binding energy (ΔG_bind_) = -7.5 kcal/mol, (Table 3). Compound **9** with the highest binding energy (-9.1 kcal/mol) docked to the target enzyme 1DXO through hydrogen bond and π–π stacking interactions with the amino acid residues Gly235, Tyr132, and Phe228 at the distances 2.33, 5.83, 4.97, 3.76, 3.63, 5.25, 4.46, and 5.97 Å, respectively. In addition, compound **12** with − 8.7 kcal/mol showed four π-cation interactions with the residue Arg272 at distances 5.36, 5.98, 5.76, and 5.94 Å, respectively (Figure 1). On the other hand, the standard drug (tropoflavin) with the binding energy − 7.5 kcal/mol binds with the target enzyme through similar amino acid residues Asn267, Arg272, and Pro264at 2.97, 2.95, 4.95, 5.95, 5.98, and 3.75 Å, respectively. The rest of compounds are shown in supplementary file section as Supplementary Figure S30.

#### Antibacterial Activity

The DNA gyrase is a topoisomerase enzyme that controls the DNA’s topological transition (Samadpour and Merrikh, 2018). In addition, the enzyme DNA gyrase has been considered as an essential for bacterial survival that catalyzes ATP-dependent negative super-coiling of bacterial chromosome (Reece et al., 1991; Tanitame et al., 2004). In this regard, in the present work, DNA gyrase has been selected as antibacterial drug target. The molecular docking simulation of the compounds **5–12** was carried out to identify their binding pattern with bacterial DNA gyrase. The compounds were observed to have the binding energies (ΔG_bind_) ranging from -9.3 to -7.7 kcal/mol; in addition the standard drug exhibited binding energy (ΔG_bind_) = -8.3 kcal/mol, as shown in Table 3. The screened compounds 5–12 docked to the target enzyme through various intermolecular interactions as hydrogen bond and π- stacking. Compound **9** has the best docking score (-9.3 kcal/mol) and exhibited four hydrogen bond interactions with the active site residues Asn46, Gly77, and Thr165 at the distances 2.76, 2.82, 3.08, and 2.29 Å, respectively. Moreover, the analogue **12** with -9.2 kcal/mol, showed intermolecular interactions through two hydrogen bond, two π-cation, and π-sigma at the distances of 3.07, 2.68, 4.39, 4.05, and 3.43 Å, respectively (Figure 2). On the other hand, the standard drug (novobiocin) with the binding energy -8.3 kcal/mol docked to the target through similar residues Arg76, His99, Ser121, Ile94, and Val97 at distances 2.94, 2.97, 2.95, 2.19, 2.06, 5.15, and 4.13 Å, respectively (Figure 2). The other docked compounds with the target enzyme are shown in Supplementary Figure S2 (Supplementary file section).

#### Structure Activity Relationship Analysis

From the obtained results, we can conclude that the compound **9** with electron donating groups like (-OH) and (-CH_3_), piperidine, and pyran moieties showed the best docking score (ΔG_bind_) toward both target enzymes NQO1 and DNA gyrase (Haredi Abdelmonsef et al., 2020; Gomha et al., 2021). Comparing the standard drugs (tropoflavin and novobiocin), it has been found that they possess the same functional groups (-OH), (-CH_3_), and pyran moieties. The docking scores of the synthesized quinoline molecules were in agreement with the experimental results which showed that the compound **9** could be used as potent inhibitor of NQO1 and DNA gyrase enzymes. Overall, the newly synthesized quinoline scaffolds have potential antioxidant and antibacterial activity and could be optimized to use as potent lead compounds as antioxidant and antibacterial agents.

#### ADMET/Pharmacokinetic Prediction Studies

*In silico* ADME/T and druglikeness prediction of the molecules **5–12**, in addition to the standard drugs (tropoflavin and novobiocin), was computationally calculated in terms of absorption, distribution, metabolic, excretion, and toxicity *via* admetSAR (Cheng et al., 2012) and Molinspiration Web-based servers. The ADME/T analysis for different synthesized molecules was found to be in acceptable ranges (Table 4). All compounds have molecular weight in the range of 279.30–405.84 g/mol (<500). The % oral intestinal drug absorption of all compounds was in the acceptable range (>80), indicating their possibilities in oral drug formulation for the treatment of bacterial infections. In addition, the new compounds exhibited little chance to cross the blood–brain barrier. The topological surface areas (TPSA) were found to be in the acceptable range (<140). In addition, H-bond acceptors (HBA) and donors (HBD) were found to be in the range of 3–6 and 2–4, respectively. Moreover, the newly synthesized compounds had high numbers of rotatable bonds (0–5), which indicates that they are flexible. Finally, the evaluation of toxicity and carcinogenic profiles for the compounds **5–12** declared that they are nontoxic and noncarcinogenic. Overall, the druglikeness study revealed that the new compounds fulfill the requirements of Lipinski’s rule of five (Ro5) (Lipinski et al., 1997), Veber (Veber et al., 2002), and Ghose (Ghose et al., 2012) without any violations, suggesting that these compounds theoretically have ideal oral bioavailability. From all these results, we can conclude that all molecules exhibited good solubility and oral bioavailability.

**TABLE 4:** List of ADME/T and physicochemical properties of standard drugs and compounds **5–12**.

	MW (g/mol)	BBB+	Caco2+	HIA+	logp	TPSA A^2^	nON	nOHNH	RBs	N violations	AMES toxicity	Carcinogenicity
Reference range	180–500	−3 to 1.2	<25 poor >500 great	<25 poor >80 high	<5	≤140	2.0–20.0	0.0–6.0	≤10		Nontoxic	Noncarcinogenic
Tropoflavin	254.24	0.50	90.57	98.5	2.97	70.67	4	2	1	0	Nontoxic	Noncarcinogenic
Novobiocin	612.63	0.82	85.84	76.04	3.93	200.02	13	6	9	2	Nontoxic	Noncarcinogenic
5a	310.35	0.95	50.00	99.5	2.68	84.58	5	3	4	0	Nontoxic	Noncarcinogenic
5b	344.80	0.95	64.25	99.4	3.34	84.58	5	3	3	0	Nontoxic	Noncarcinogenic
5c	344.80	0.95	56.09	99.4	3.34	84.58	5	3	3	0	Nontoxic	Noncarcinogenic
5d	324.38	0.95	55.64	99.5	2.99	84.58	5	3	3	0	Nontoxic	Noncarcinogenic
6	279.30	0.97	57.21	98.81	2.07	87.38	4	4	0	0	Nontoxic	Noncarcinogenic
7	279.30	0.94	57.85	98.91	3.14	58.56	3	2	0	0	Nontoxic	Noncarcinogenic
9	371.39	0.95	52.00	99.19	4.57	70.00	4	2	1	0	Nontoxic	Noncarcinogenic
10	405.84	0.97	60.53	96.95	4.92	79.53	5	2	3	0	Nontoxic	Noncarcinogenic
11	400.48	0.96	51.54	99.50	4.28	75.79	5	2	5	0	Nontoxic	Noncarcinogenic
12	372.38	0.96	79.08	99.27	3.47	96.02	6	3	1	0	Nontoxic	Noncarcinogenic

The pharmacokinetic and physicochemical properties of the molecules (**5–12**). The agreeable ranges are as follows: Mol wt.: (<500); %Human oral absorption: >80% high, <25% low. logp, logarithm of partition coefficient between n-octanol and water <5; TPSA, topological polar surface area ≤140; nON, number of hydrogen bond acceptors 2.0–20.0; nOHNH, number of hydrogen bond donors 0.0–6.0; RBs, number of rotatable bonds ≤10.

## Conclusion

In conclusion, we described a rapid, efficient, and low-cost method for synthesis of some quinoline analogues by using four components under solvent-free conditions. In addition, all synthesized compounds were *in vitro* screened for their antioxidant and antibacterial activity. Further, *in silico* molecular docking studies were achieved to support the biological experiments. The compound **9** displayed promising antioxidant and antibacterial activity, which was well supported by the *in silico* binding score, which showed it to have the highest binding energy of -9.1 and -9.3 kcal/mol against the target enzymes 1DXO and 1AJ6, respectively. In addition, compound **9** obeyed the Lipinski’s rule of five, Veber, and Ghose . The experimental and *in silico* findings indicated that compound **9** could be used as a promising inhibitor of enzymes NQO1 and DNA gyrase.

## Data Availability

The original contributions presented in the study are included in the article/Supplementary Material; further inquiries can be directed to the corresponding author.

## References

[B1] Abdelmonsef A. H. (2019). Computer-Aided Identification of Lung Cancer Inhibitors through Homology Modeling and Virtual Screening. Egypt. J. Med. Hum. Genet. 20, 1–14. 10.1186/s43042-019-0008-3

[B2] Abdelmonsef A. H. Dulapalli R. Dasari T. Padmarao L. S. Mukkera T. Vuruputuri U. (2016). Identification of Novel Antagonists for Rab38 Protein by Homology Modeling and Virtual Screening. Comb Chem High Throughput Screen. 19 (10). 10.2174/1386207319666161026153237 27784220

[B3] Abdelmonsef A. H. Mosallam A. M. (2020). Synthesis, *In Vitro* Biological Evaluation and In Silico Docking Studies of New Quinazolin‐2,4‐dione Analogues as Possible Anticarcinoma Agents. J. Heterocycl Chem. 57, 1637–1654. 10.1002/jhet.3889

[B4] Acharyulu P. V. R. Dubey P. K. Reddy P. V. V. P. Suresh T. (2008). Synthesis of New 4(3H)-Quinazolinone Derivatives under Solvent-free Conditions Using PEG-400. Arkivoc. 2008 (11), 104–111. 10.3998/ark.5550190.0009.b10

[B5] Asif M. (2014). Chemical Characteristics, Synthetic Methods, and Biological Potential of Quinazoline and Quinazolinone Derivatives. Int. J. Med. Chem. 2014, 1–27. 10.1155/2014/395637 PMC4321853 25692041

[B6] Atia A. Abdullah A. (2020). NQO1 Enzyme and its Role in Cellular Protection; an Insight. Iberoamerican J. Med. 02, 306–313. 10.5281/zenodo.3877528

[B7] Bauer A. W. Kirby W. M. M. Sherris J. C. Turck M. (1966). Antibiotic Susceptibility Testing by a Standardized Single Disk Method. Am. J. Clin. Pathol. 45 (4), 493–496. 10.1308/rcsann.2013.95.7.532 5325707

[B8] Bawa S. Kumar S. Drabu S. Kumar R. (2010). Structural Modifications of Quinoline-Based Antimalarial Agents: Recent Developments. J. Pharm. Bioall Sci. 2 (2), 64. 10.4103/0975-7406.67002 PMC3147106 21814435

[B9] Cheng F. Li W. Zhou Y. Shen J. Wu Z. Liu G. (2012). AdmetSAR: A Comprehensive Source and Free Tool for Assessment of Chemical ADMET Properties. J. Chem. Inf. Model. 52 (11), 3099–3105. 10.1021/ci300367a 23092397

[B10] Dallakyan S. Olson A. J. (2015). Small-Molecule Library Screening by Docking with PyRx. Chem. Biol. 1263, 243–250. 10.1016/B978-0-12-394447-4.10004-510.1007/978-1-4939-2269-7_19 25618350

[B11] Dasari T. Kondagari B. Dulapalli R. Abdelmonsef A. H. Mukkera T. Padmarao L. S. (2017). Design of Novel lead Molecules against RhoG Protein as Cancer Target - a Computational Study. J. Biomol. Struct. Dyn. 35 (14), 3119–3139. 10.1080/07391102.2016.1244492 27691842

[B26] Dinkova-Kostova A. T. Talalay P. (2010). NAD(P)H:quinone acceptor oxidoreductase 1 (NQO1), a multifunctional antioxidant enzyme and exceptionally versatile cytoprotector Arch. Biochem. Biophys. 501 (1), 116–123. 10.1016/j.abb.2010.03.019 20361926 PMC2930038

[B12] Dua R. Shrivastava S. Sonwane S. K. Srivastava S. K. (2011). Pharmacological Significance of Synthetic Heterocycles Scaffold : A Review. Advan. Biol. Res. 5 (3), 120–144. 10.1023/B:SOLA.0000013030.09729.38

[B13] El-Maghraby A. M. Aboubakr H. A. (2019). Synthesis, Characterization and Insilico Molecular Docking Studies of Novel Chromene Derivatives as Rab23 Inhibitors. Egypt. J. Chem. 63 (4), 1341–1358. 10.21608/ejchem.2019.15013.1911

[B14] El-Maghraby A. M. (2014). Green Chemistry: New Synthesis of Substituted Chromenes and Benzochromenes *via* Three-Component Reaction Utilizing Rochelle Salt as Novel Green Catalyst. Org. Chem. Int. 2014 (Scheme 1), 1–6. 10.1155/2014/715091

[B15] Faig M. Bianchet M. A. Talalay P. Chen S. Winski S. Ross D. (2000). Structures of Recombinant Human and Mouse NAD(P)H:Quinone Oxidoreductases: Species Comparison and Structural Changes with Substrate Binding and Release. Proc. Natl. Acad. Sci. 97 (7), 3177–3182. 10.1073/pnas.97.7.3177 10706635 PMC16212

[B16] Ghose A. K. HerbertzHudkins T. Hudkins R. L. Dorsey B. D. Mallamo J. P. (2012). Knowledge-Based, Central Nervous System (CNS) Lead Selection and Lead Optimization for CNS Drug Discovery. ACS Chem. Neurosci. 3 (1), 50–68. 10.1021/cn200100h 22267984 PMC3260741

[B17] Gómez C. M. M. Vladimir V. K. (2013). “Recent Developments on Antimicrobial Quinoline Chemistry,” in Microbial Pathogens and Strategies for Combating Them: Science, Technology and Education. Editor Méndez-Vilas A. (FORMATEX), December, 666–677.

[B18] Gomha S. M. Hyam A. A. Doaa Zh H. Aboubakr H. A. Mohamed E-N. (2021). Thiazole-Based Thiosemicarbazones: Synthesis, Cytotoxicity Evaluation and Molecular Docking Study. Drug Des Devel Ther. Vol. 15 (February), 659–677. 10.2147/DDDT.S291579 PMC7900779 33633443

[B19] Ha A. Sp L. (2016). Human Rab8b Protein as a Cancer Target - an In Silico Study. J. Comput. Sci. Syst. Biol. 9 (4). 10.4172/jcsb.1000231

[B20] Haredi Abdelmonsef A. Eldeeb Mohamed M. El-Naggar M. Temairk H. Mohamed Mosallam A. (2020). Novel Quinazolin-2,4-Dione Hybrid Molecules as Possible Inhibitors against Malaria: Synthesis and In Silico Molecular Docking Studies. Front. Mol. Biosci. 7, 105. 10.3389/fmolb.2020.00105 32582763 PMC7291371

[B21] Holdgate G. A. Tunnicliffe A. Ward W. H. J. Weston S. A. Rosenbrock G. Barth P. T. (1997). The Entropic Penalty of Ordered Water Accounts for Weaker Binding of the Antibiotic Novobiocin to a Resistant Mutant of DNA Gyrase: A Thermodynamic and Crystallographic Study. Biochemistry 36 (32), 9663–9673. 10.1021/bi970294+ 9245398

[B22] Hussein M. A. Ola H. Z. Abo-bakr H. A. M. Rizk S. A. Shaimaa M. Amany S. K. (2018). Synthesis, Molecular Docking and Insecticidal Activity Evaluation of Chromones of Date Palm Pits Extract against Culex Pipiens (Diptera: Culicidae). Int. J. Mosquito Res. 5 (4), 22–32.

[B23] Kumar S. Bawa S. Gupta H. (2009). Biological Activities of Quinoline Derivatives. Mini Rev Med Chem. 9 (14), 1648–1654. 10.2174/138955709791012247 20088783

[B24] Lipinski C. A. Lombardo F. Dominy B. W. Feeney P. J. (1997). Experimental and Computational Approaches to Estimate Solubility and Permeability in Drug Discovery and Develop Ment Settings. Adv. Drug Deliv. Rev. 23 (August), 3–25. 10.1016/S0169-409X(00)00129-0 11259830

[B25] Luo Z. G. Cheng C. Z. Fang W. Hong Q. H. Cun X. W. Hong G. D. (2009). Synthesis and Biological Activities of Quinoline Derivatives as HIV-1 Integrase Inhibitors. Chem. Res. Chin. Universities 25 (6), 841–845.

[B27] Meshram H. M. Chennakesava Reddy B. Aravind Kumar D. Kalyan M. Ramesh P. Kavitha P. (2012). Synthesis and Cytotoxicity of New Quinoline Derivatives. Indian J. Chem. - Section B Org. Med. Chem. 51 (9), 1411–1416.

[B28] Noser A. A. El-Naggar M. Donia T. Abdelmonsef A. H. (2020). Synthesis, In Silico and *In Vitro* Assessment of New Quinazolinones as Anticancer Agents *via* Potential AKT Inhibition. Molecules 25 (20), 4780. 10.3390/molecules25204780 PMC7594071 33080996

[B29] O’Boyle N. M. Michael B. Craig A. J. Chris M. Tim V. Geoffrey R. H. (2011). Open Babel: An Open Chemical Toolbox. J. Cheminformatics 3 (10), 33. 10.1186/1758-2946-3-33 PMC3198950 21982300

[B30] Palming J. Sjöholm K. Jernås M. Lystig T. C. Gummesson A. Romeo S. (2007). The Expression of NAD(P)H:Quinone Oxidoreductase 1 Is High in Human Adipose Tissue, Reduced by Weight Loss, and Correlates with Adiposity, Insulin Sensitivity, and Markers of Liver Dysfunction. Dysfunction 92 (6), 2346–2352. 10.1210/jc.2006-2476 17405841

[B31] Prieto P. Pineda M. Aguilar M. (1999). Spectrophotometric Quantitation of Antioxidant Capacity through the Formation of a Phosphomolybdenum Complex: Specific Application to the Determination of Vitamin E. Anal. Biochem. 269 (2), 337–341. 10.1006/abio.1999.4019 10222007

[B32] Rashdan H. R. M. Rashdan H. R. M. Aboubakr H. A. Ihsan A. S. Sobhi M. G. Abdel Mohsen M. S. (2020). Synthesis, Molecular Docking Screening and Anti-proliferative Potency Evaluation of Some New Imidazo[2,1-b]Thiazole Linked Thiadiazole Conjugates. Molecules 25 (21), 4997. 10.3390/molecules25214997 PMC7663531 33126630

[B33] Reece R. J. Maxwell A. Wang James. C. (1991). DNA Gyrase: Structure and Function. Crit. Rev. Biochem. Mol. Biol. 26 (3–4), 335–375. 10.3109/10409239109114072 1657531

[B34] Rondla R. PadmaRao L. S. Ramatenki V. Haredi-Abdel-Monsef A. Potlapally S. R. Vuruputuri U. (2017). Selective ATP Competitive Leads of CDK4: Discovery by 3D-QSAR Pharmacophore Mapping and Molecular Docking Approach. Comput. Biol. Chem. 71 (December), 224–229. 10.1016/j.compbiolchem.2017.11.005 29153893

[B35] Samadpour A. N. Merrikh H. (2018). DNA Gyrase Activity Regulates DnaA-dependent Replication Initiation inBacillus Subtilis. Mol. Microbiol. 108 (2), 115–127. 10.1111/mmi.13920 29396913 PMC5893406

[B36] Sangani C. Shah N. Patel M. Patel R. (2012). Microwave Assisted Synthesis of Novel 4h-Chromene Derivatives Bearing Phenoxypyrazole and Their Antimicrobial Activity Assess. Jscs. 77 (9), 1165–1174. 10.2298/jsc120102030s

[B37] Shang X.-F. Morris-Natschke S. L. Liu Y.-Q. Guo X. Xu X.-S. Goto M. (2018a). Biologically Active Quinoline and Quinazoline Alkaloids Part I. Med. Res. Rev. 38, 775–828. 10.1002/med.21466 28902434 PMC6421866

[B38] Shang X.-F. Morris-Natschke S. L. Liu Y.-Q. Guo X. Xu X.-S. Goto M. (2018b). Biologically Active Quinoline and Quinazoline Alkaloids Part II. Med. Res. Rev. 38, 1614–1660. 10.1002/med.21492 29485730 PMC6105521

[B39] Shehadi I. A. Rashdan H. R. M. Abdelmonsef A. H. (2020). Homology Modeling and Virtual Screening Studies of Antigen MLAA-42 Protein: Identification of Novel Drug Candidates against Leukemia-An In Silico Approach. Comput. Math. Methods Med. 2020, 1–12. 10.1155/2020/8196147 PMC7102452 32256683

[B40] Sidoryk K. Świtalska M. Jaromin A. Cmoch P. Bujak I. Kaczmarska M. (2015). The Synthesis of Indolo[2,3-b]Quinoline Derivatives with a Guanidine Group: Highly Selective Cytotoxic Agents. Eur. J. Med. Chem. 105, 208–219. 10.1016/j.ejmech.2015.10.022 26496013

[B41] Tanitame A. Oyamada Y. Ofuji K. Fujimoto M. Iwai N. Hiyama Y. (2004). Synthesis and Antibacterial Activity of a Novel Series of Potent DNA Gyrase Inhibitors. Pyrazole Derivatives. J. Med. Chem. 47 (14), 3693–3696. 10.1021/jm030394f 15214796

[B42] Veber D. F. Stephen R. J. Hung-Yuan C. Brian R. S. Keith W. W. Kenneth D. (2002). Molecular Properties that Influence the Oral Bioavailability of Drug Candidates. J. Med. Chem. 45 (12), 2615–2623. 10.1021/jm020017n 12036371

[B43] Wan C. Yu Y. Zhou S. Liu W. Tian S. Cao S. (2011). Antioxidant Activity and Free Radical-Scavenging Capacity of Gynura Divaricata Leaf Extracts at Different Temperatures. Pharmacogn Mag. 7, 40–45. 10.4103/0973-1296.75900 21472078 PMC3065156

[B44] Wang H.-X. Liu H.-Y. Li W. Zhang S. Wu Z. Li X. (2019). Design, Synthesis, Antiproliferative and Antibacterial Evaluation of Quinazolinone Derivatives. Med. Chem. Res. 28 (2), 203–214. 10.1007/s00044-018-2276-8

