# Supplementary material for: Eco-Friendly Synthesis, Biological Evaluation, and In Silico Molecular Docking Approach of Some New Quinoline Derivatives as Potential Antioxidant and Antibacterial Agents
Source: Front Chem. 2021 Jun 10;9:679967. doi: 10.3389/fchem.2021.679967 (PMC8222571; doi:10.3389/fchem.2021.679967)
Supplement: Supplementary file 1 [file DataSheet1.PDF]

Supplementary file

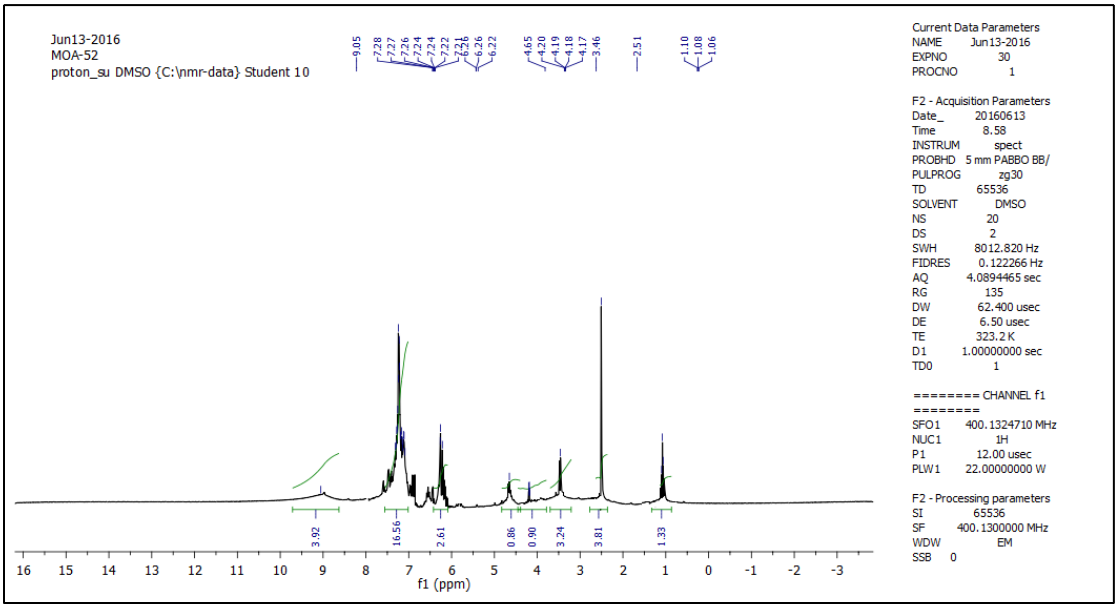

Figure S1. ^{1}H-NMR spectrum of compound 5a

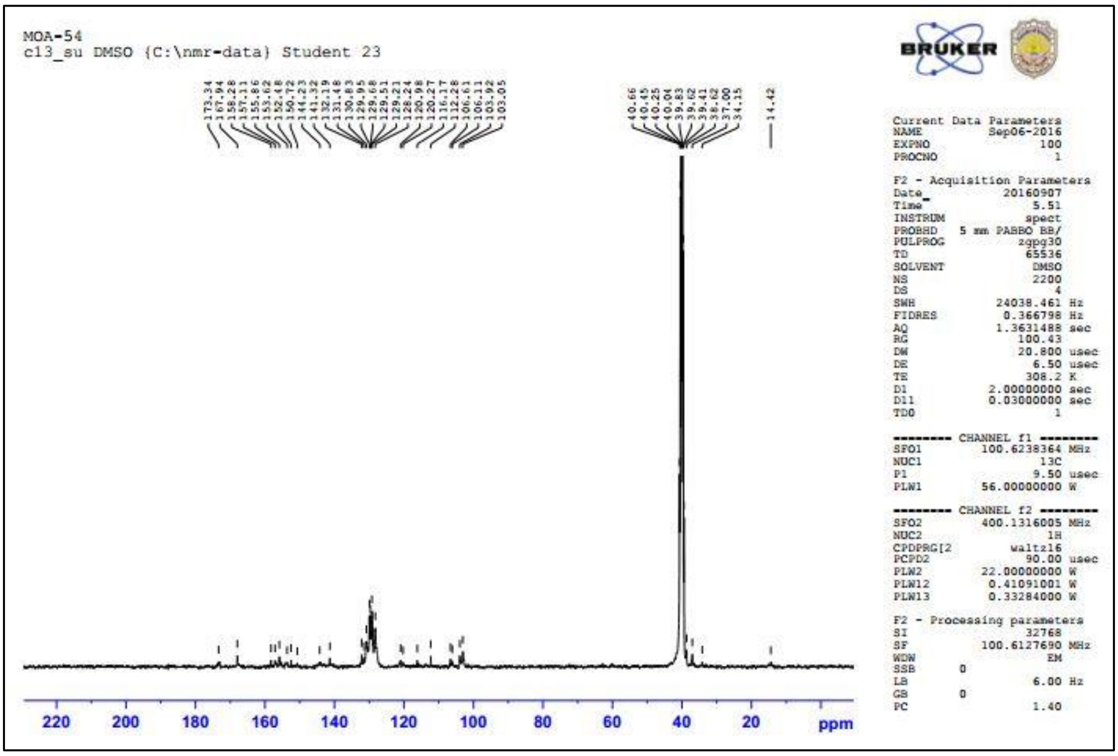

Figure S2. ^{13}C-NMR spectrum of compound 5a

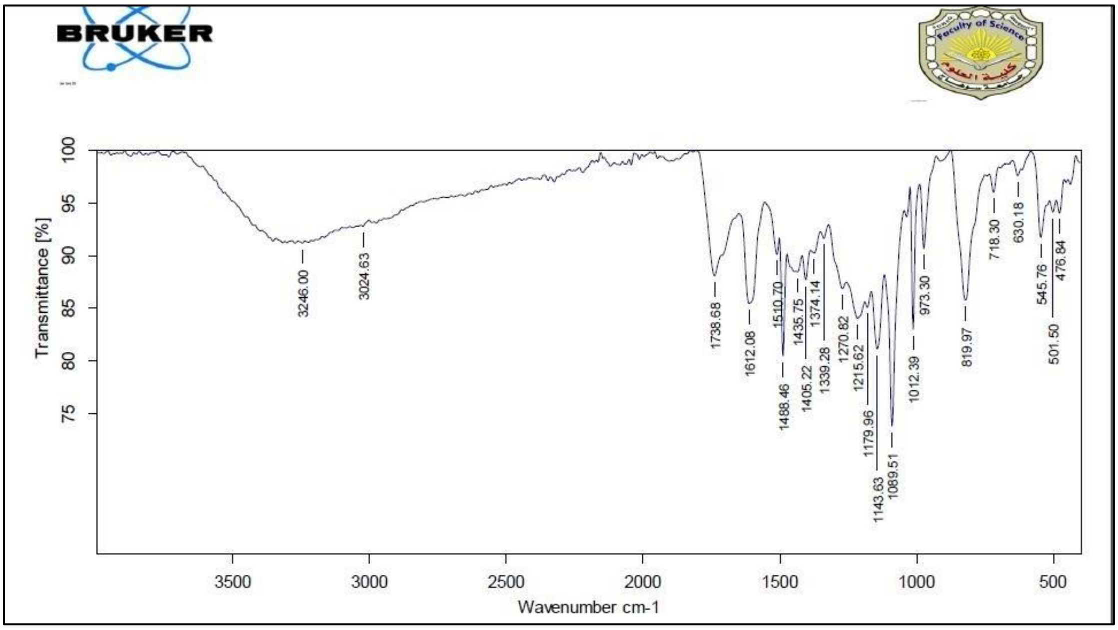

Figure S3. IR spectrum of compound 5b

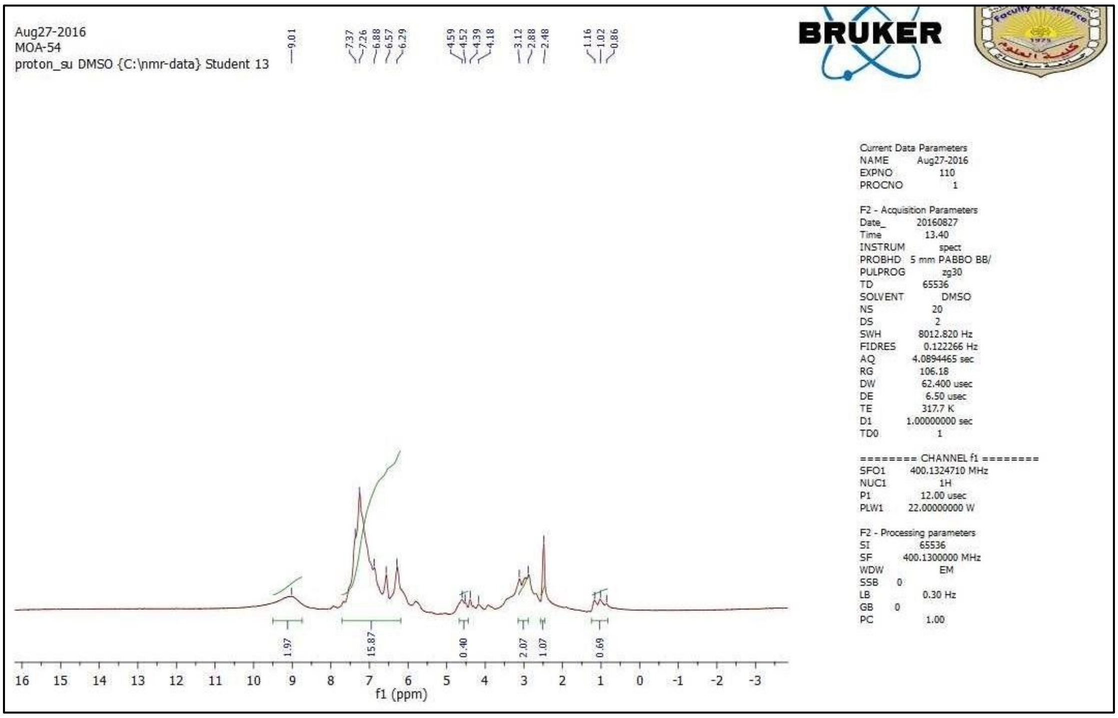

Figure S4. ^{1}H-NMR spectrum of compound 5b

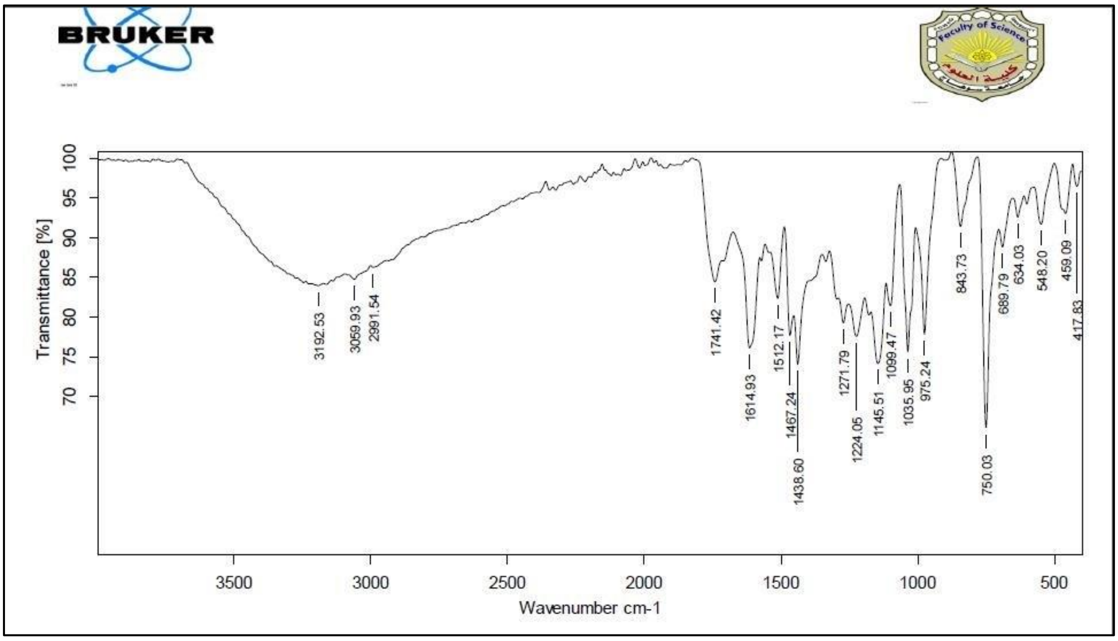

Figure S5. IR spectrum of compound 5c

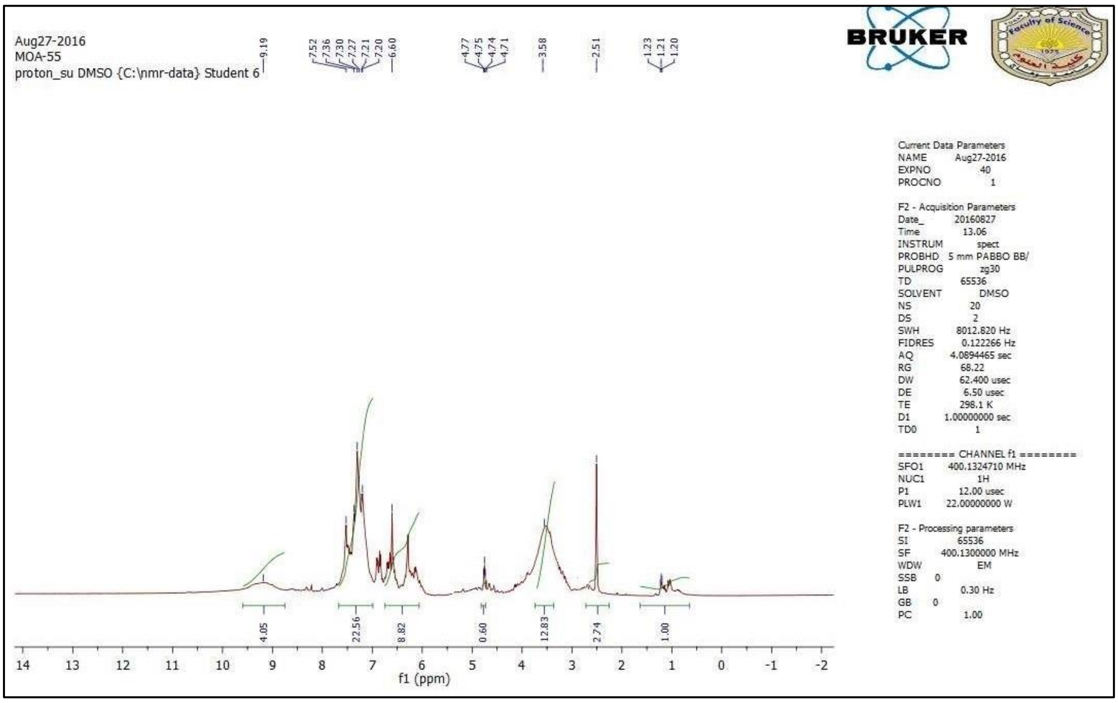

Figure S6. ^{1}H-NMR spectrum of compound 5c

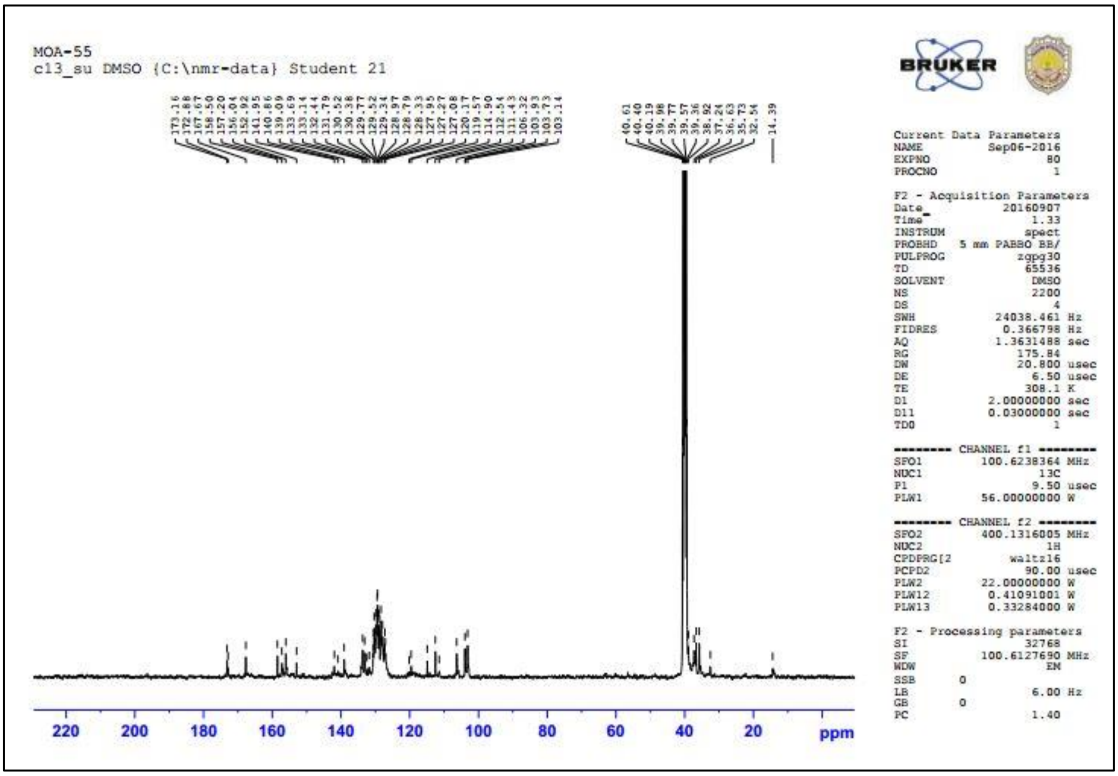

Figure S7. ^{13}C-NMR spectrum of compound 5c

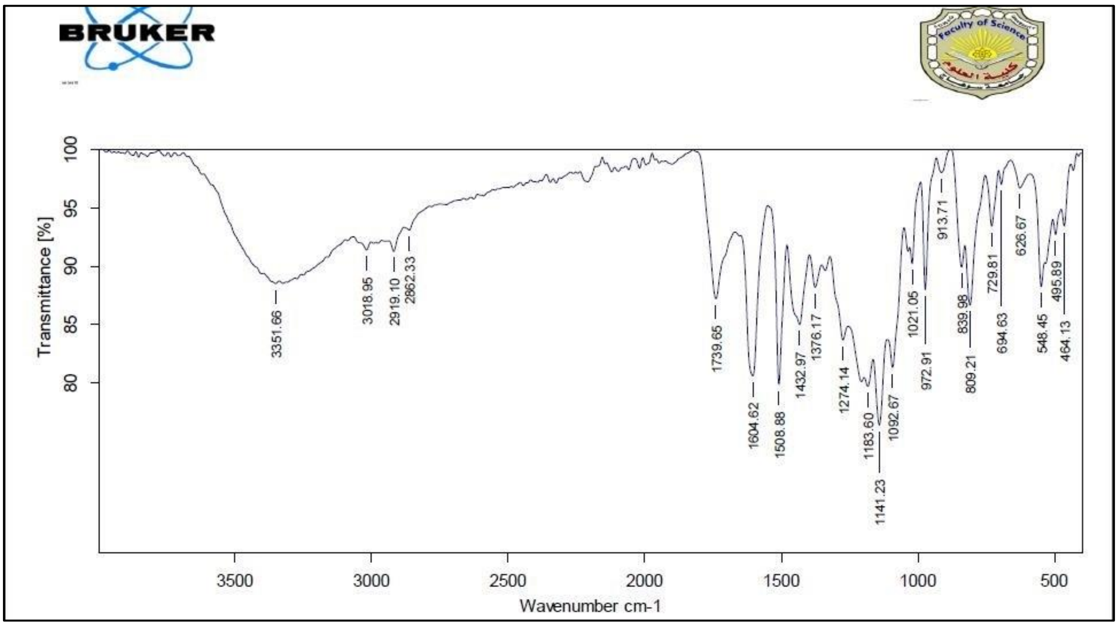

Figure S8. IR spectrum of compound 5d

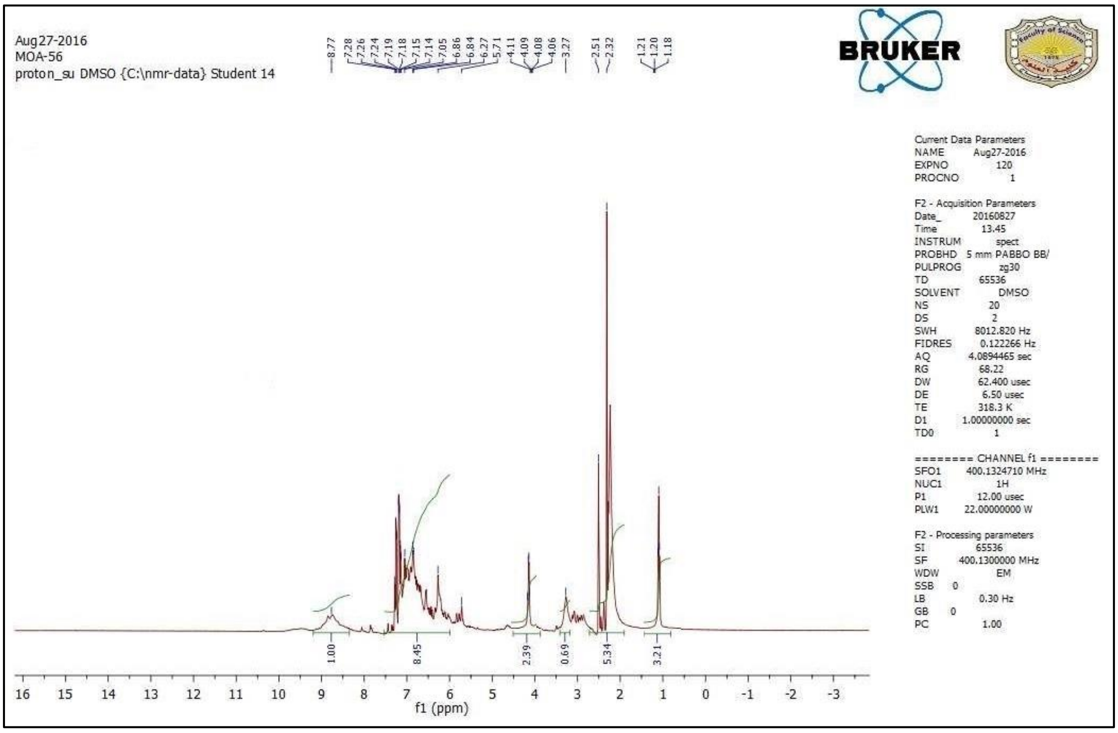

Figure S9. ^{1}H-NMR spectrum of compound 5d

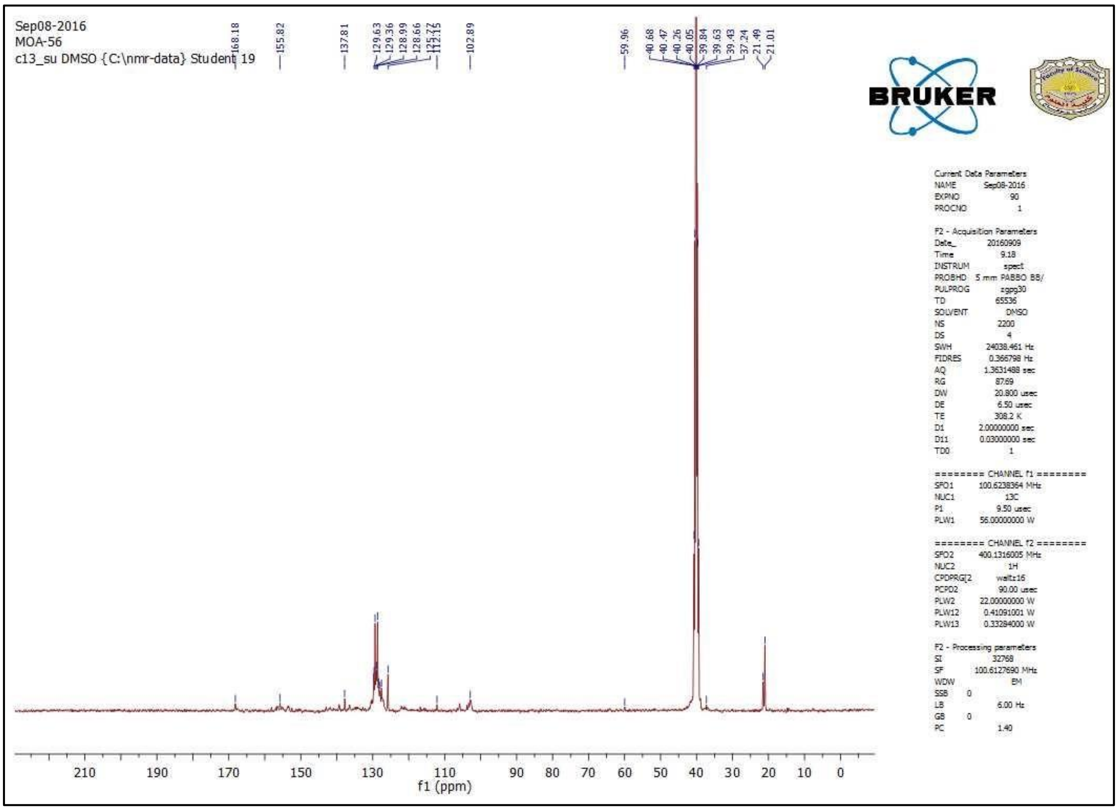

Figure S10. ^{13}C-NMR spectrum of compound 5d

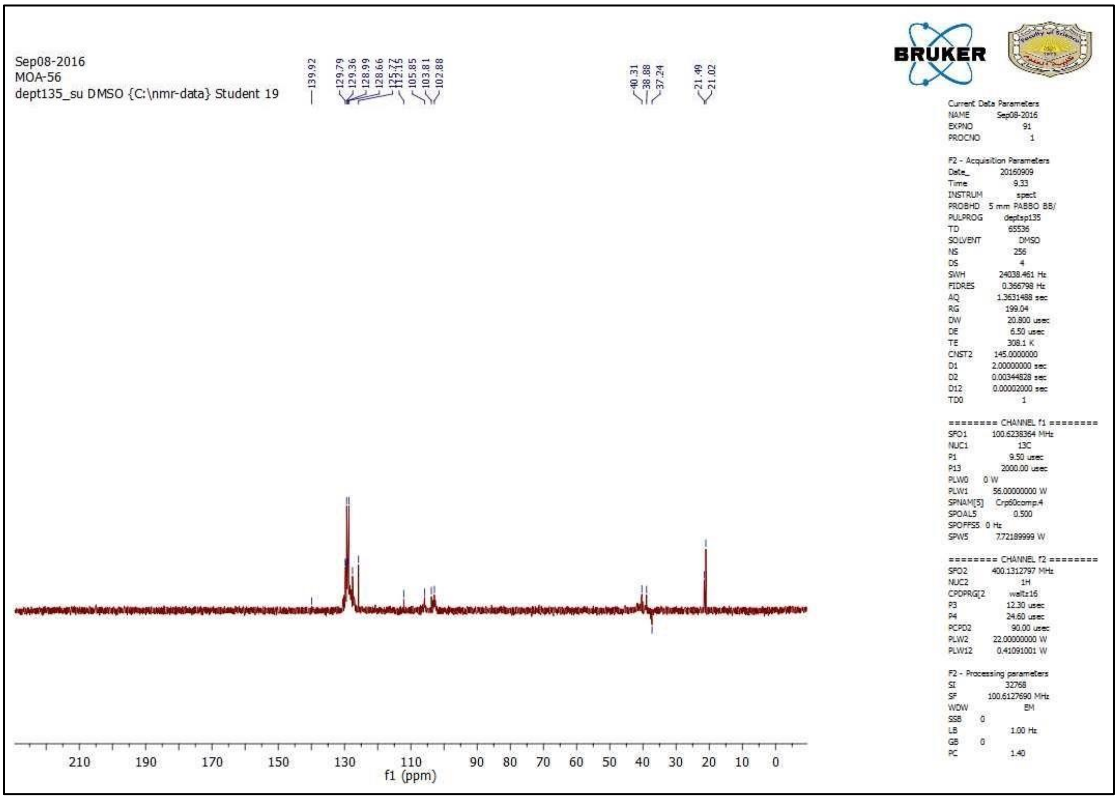

Figure S11. DEPT spectrum of compound 5d

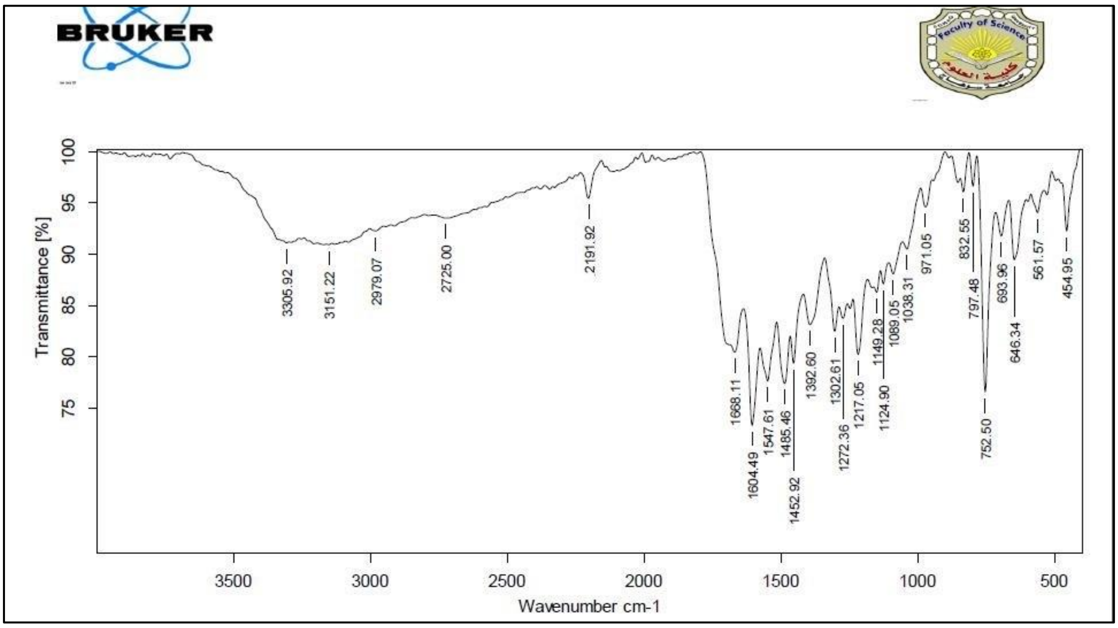

Figure S12. IR spectrum of compound 6

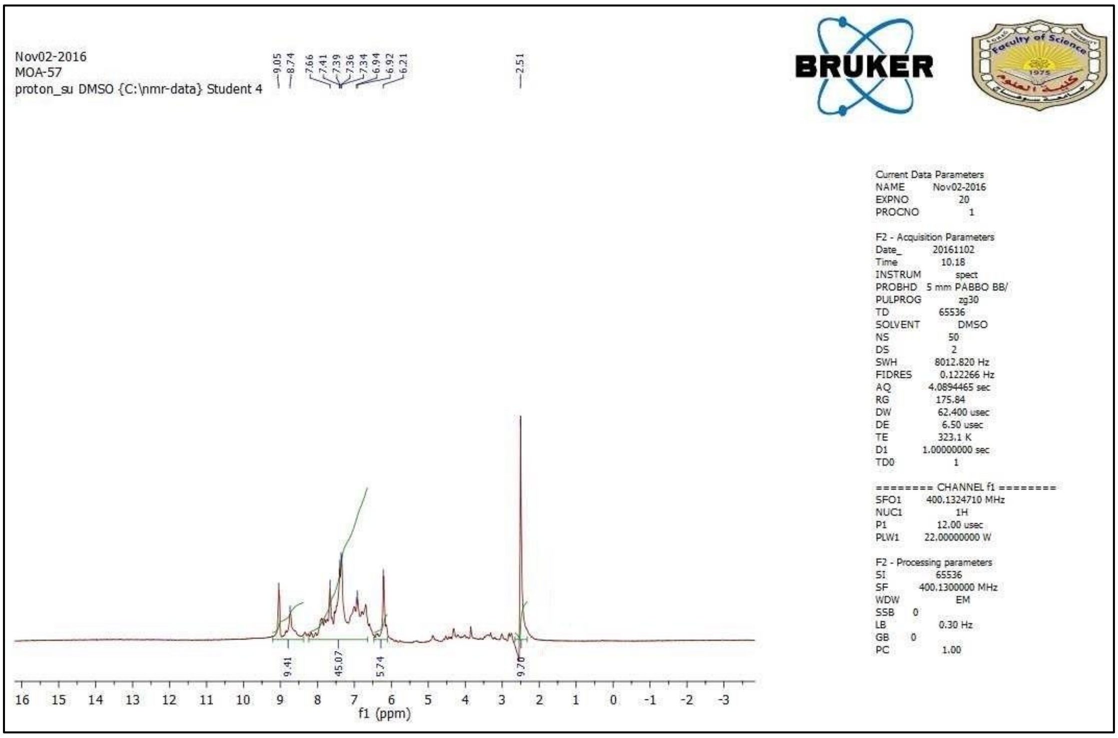

Figure S13.^{1}H-NMR spectrum of compound 6

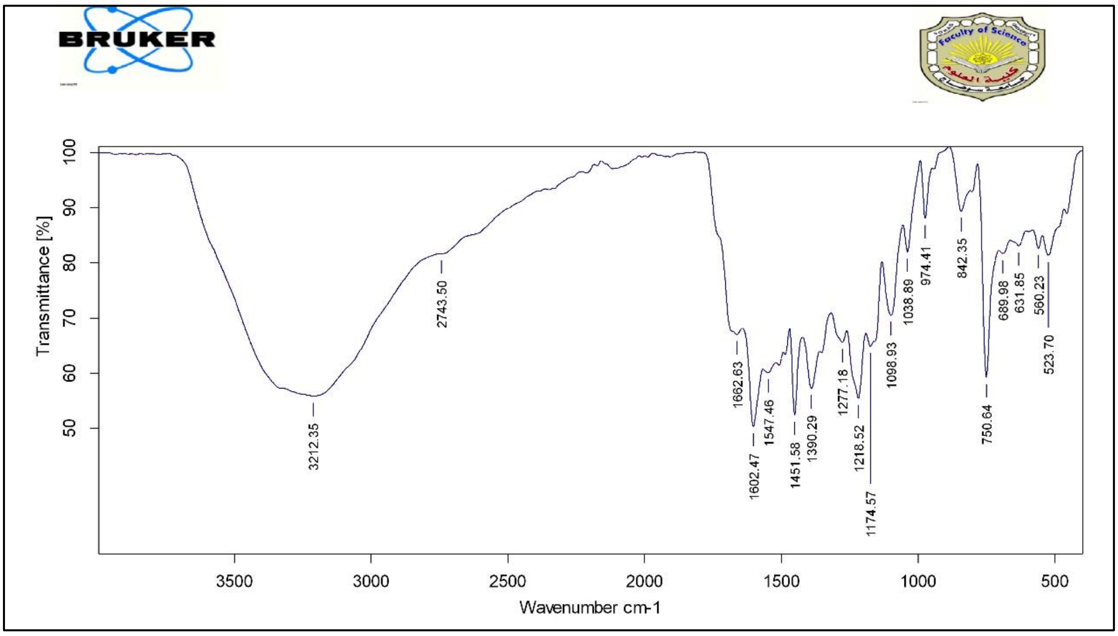

Figure S14. IR spectrum of compound 7

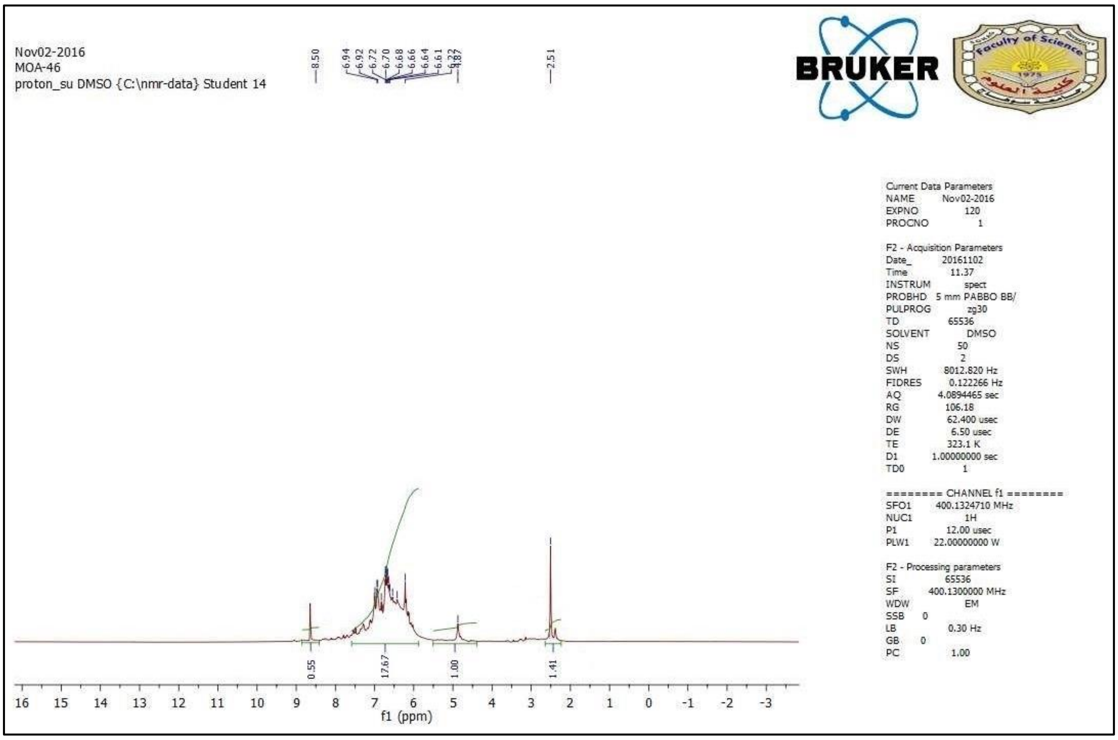

Figure S15. ^{1}H-NMR spectrum of compound 7

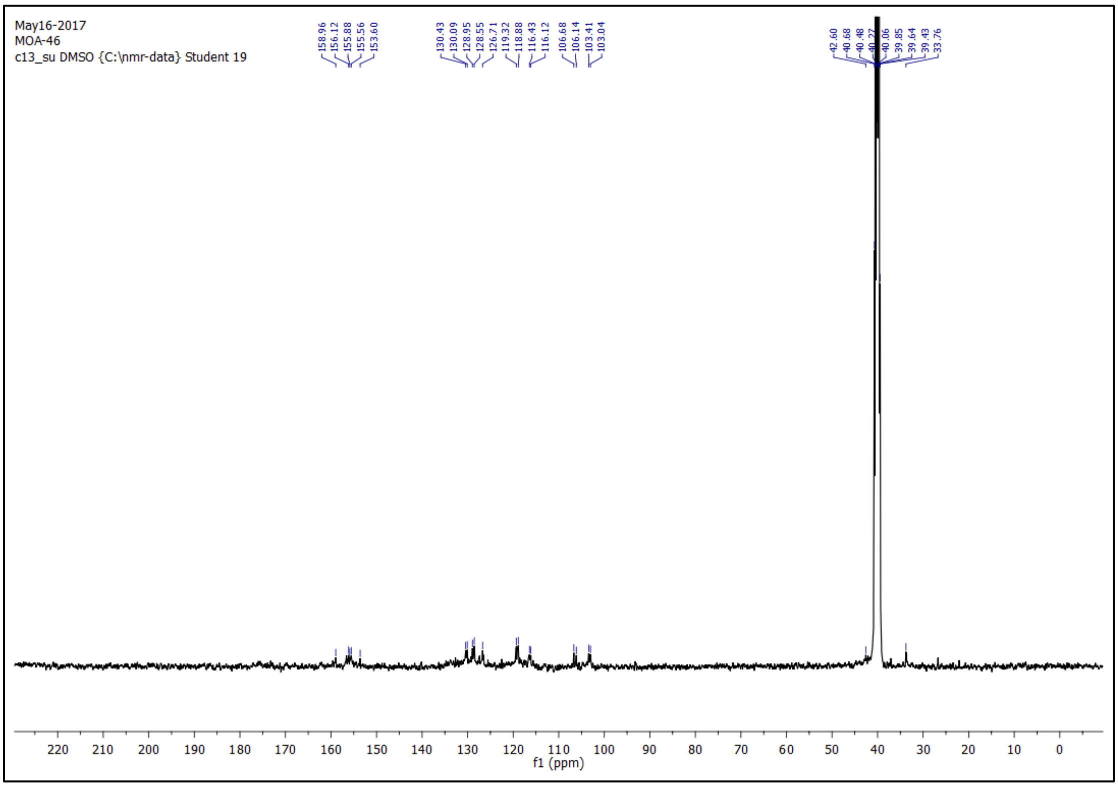

Figure S16.^{13}C-NMR spectrum of compound 7

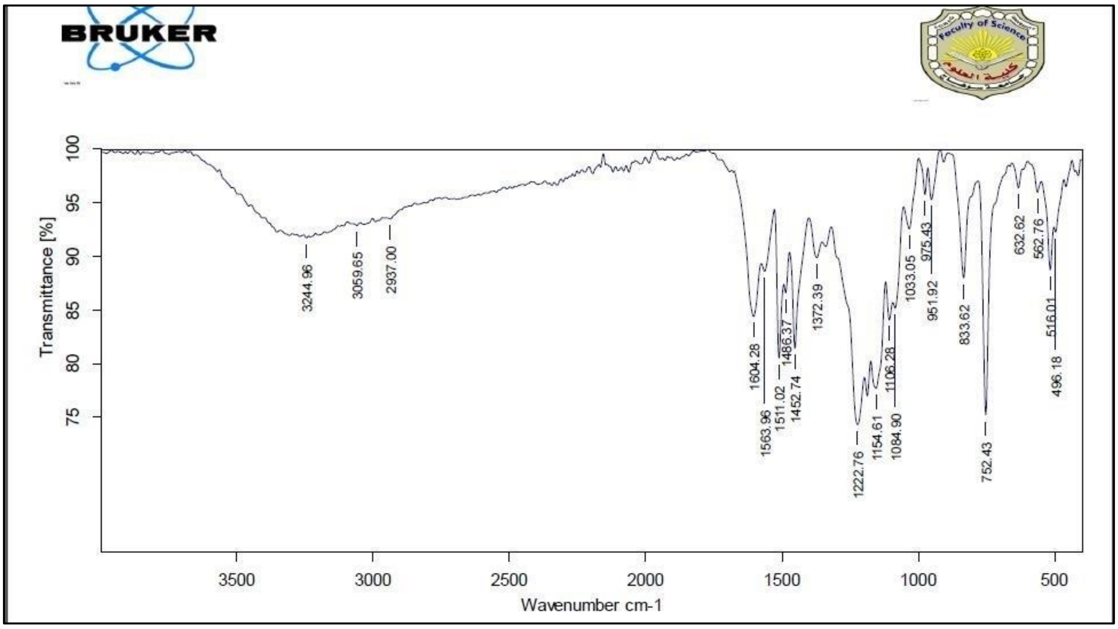

Figure S17. IR spectrum of compound 9

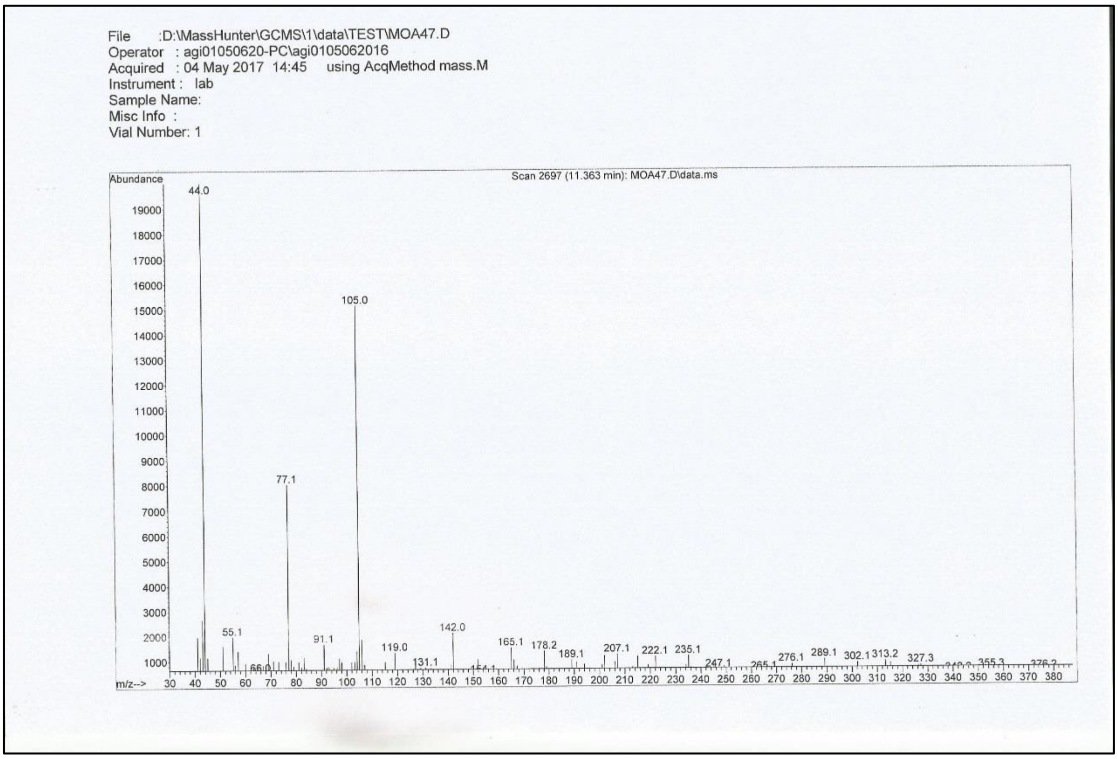

Figure S18. Mass spectrum of compound 9

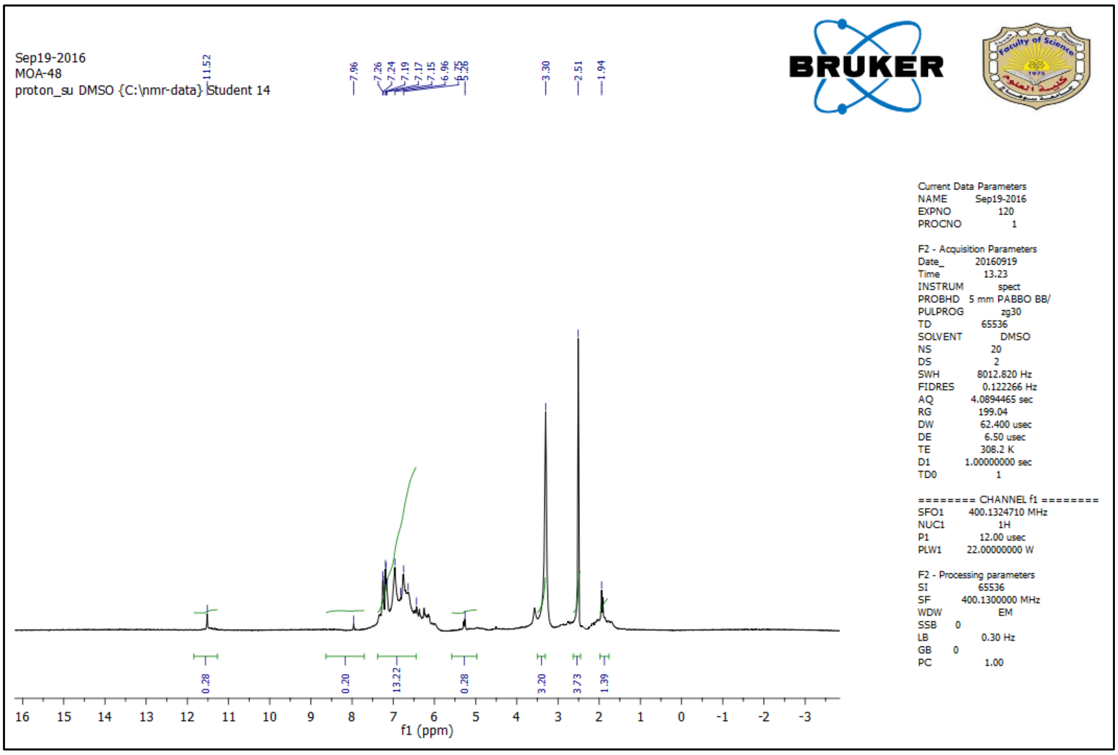

Figure S19. ^{1}H-NMR spectrum of compound 9

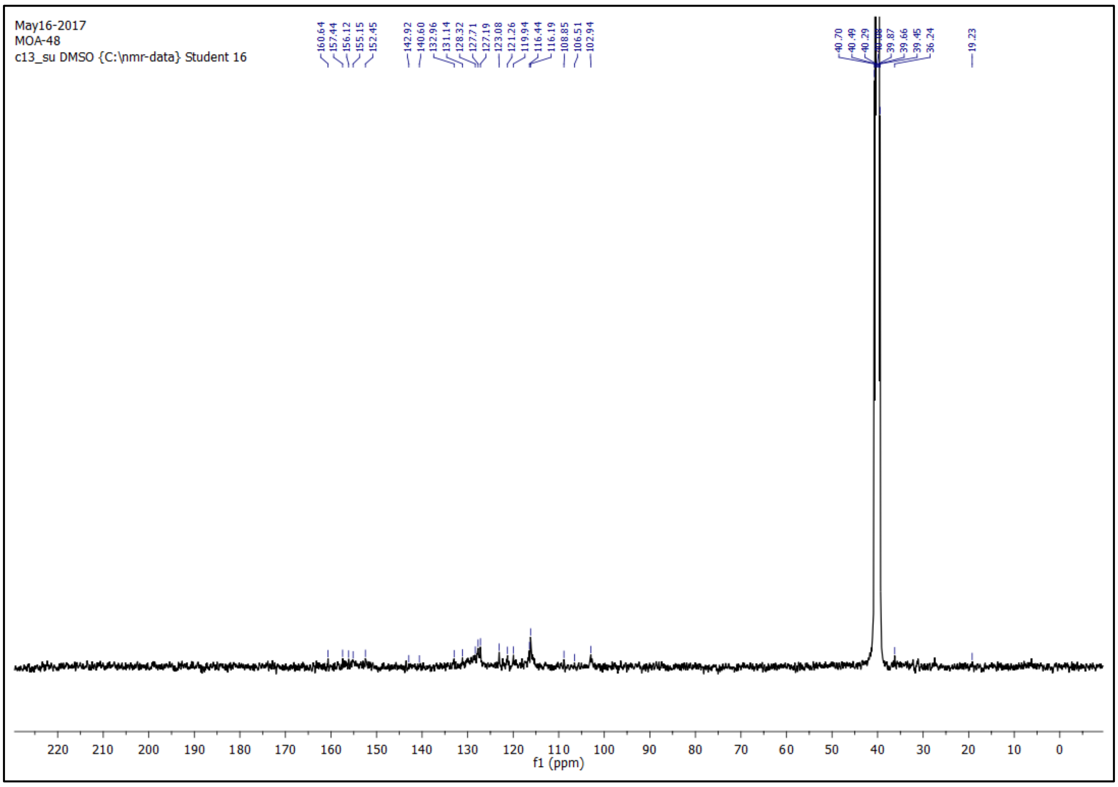

Figure S20. ^{13}C-NMR spectrum of compound 9

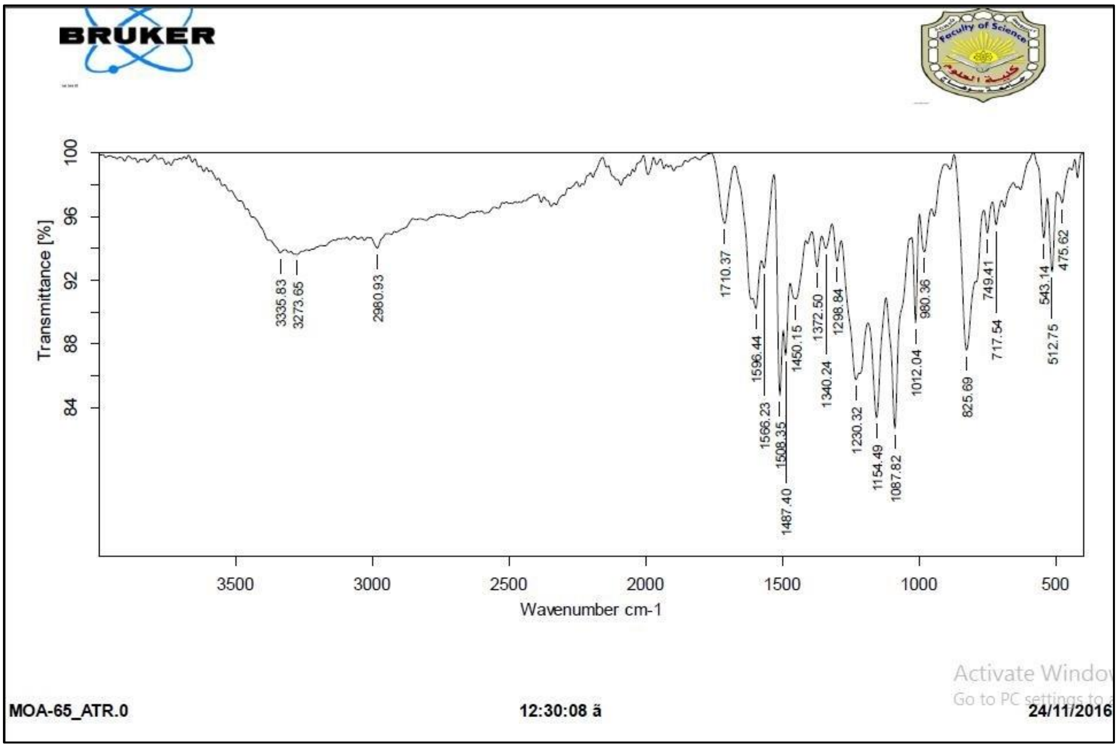

Figure S21. IR spectrum of compound 10

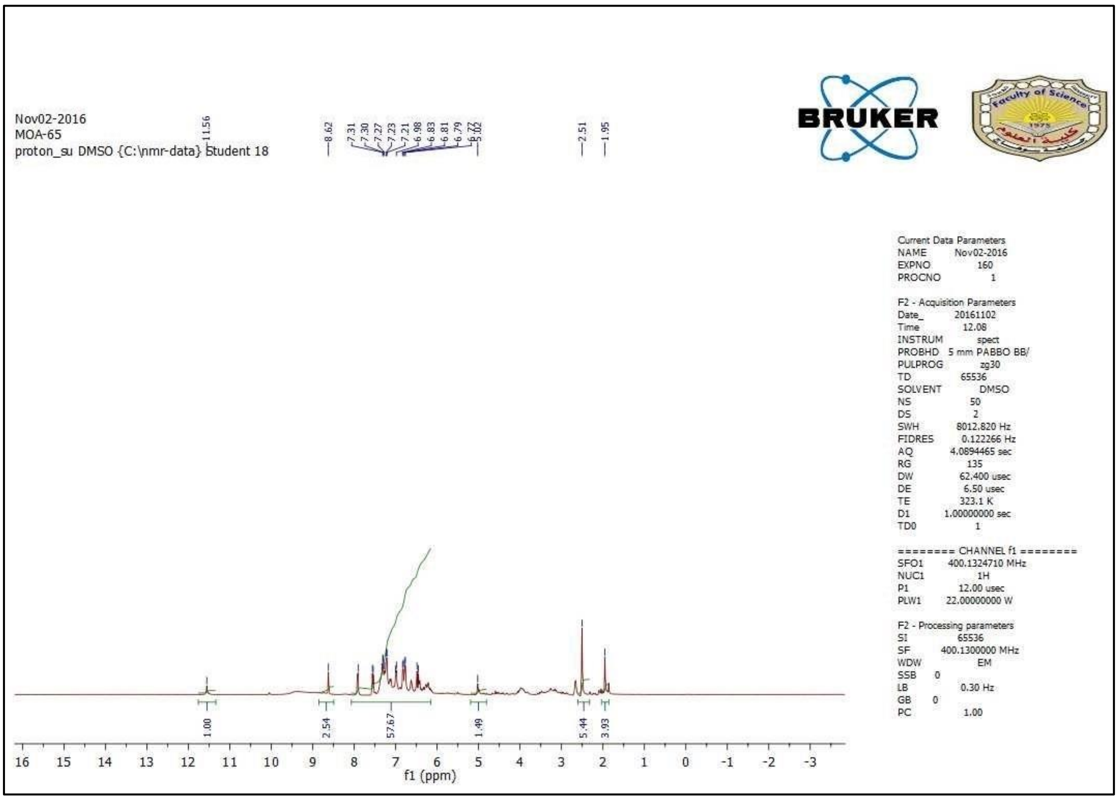

Figure S22. ^{1}H-NMR spectrum of compound 10

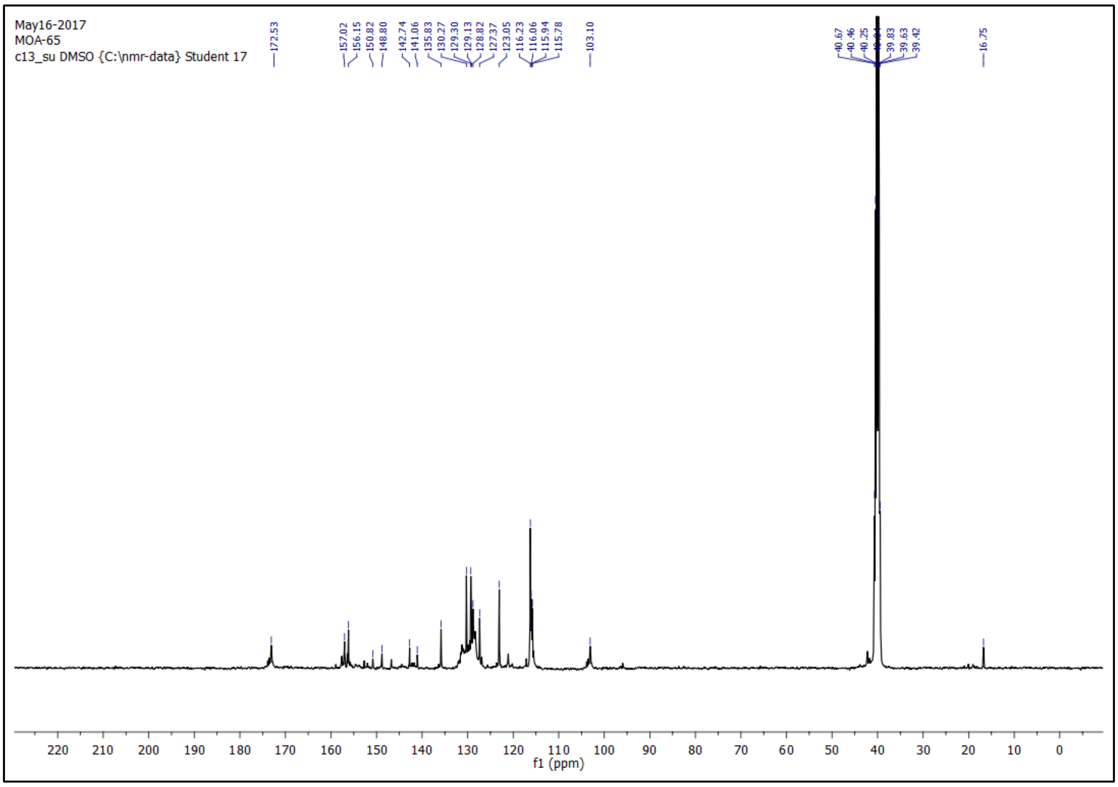

Figure S23. ^{13}**C-NMR spectrum of compound 10**

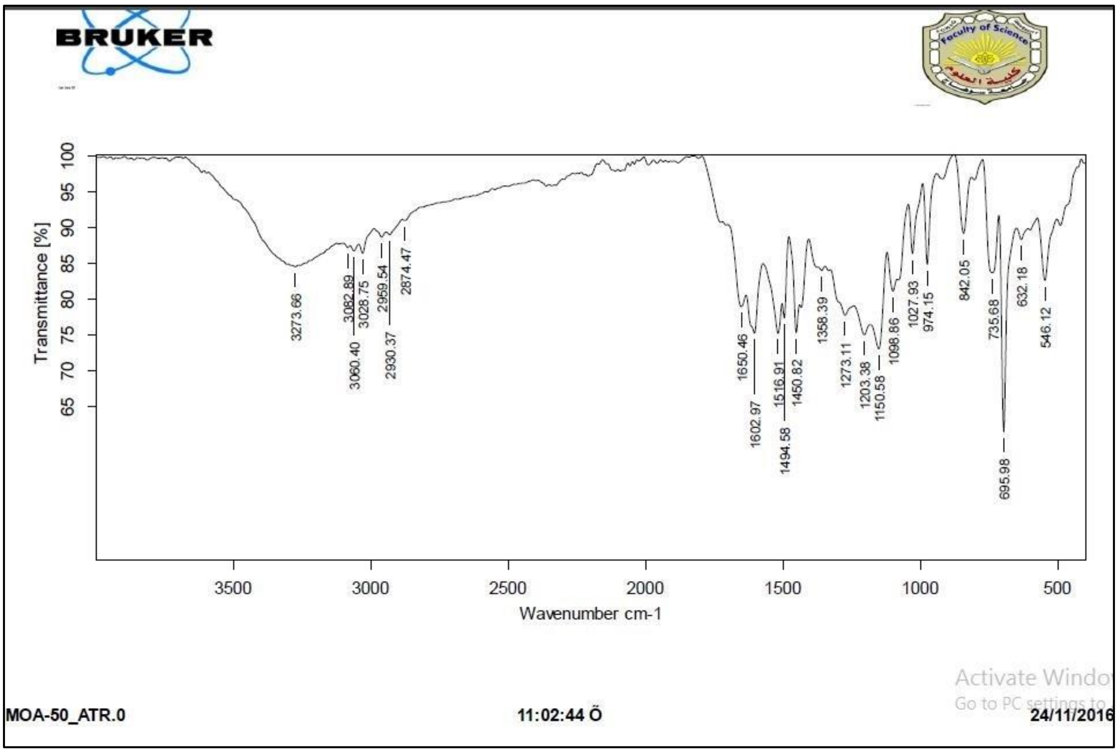

Figure S24. IR spectrum of compound 11

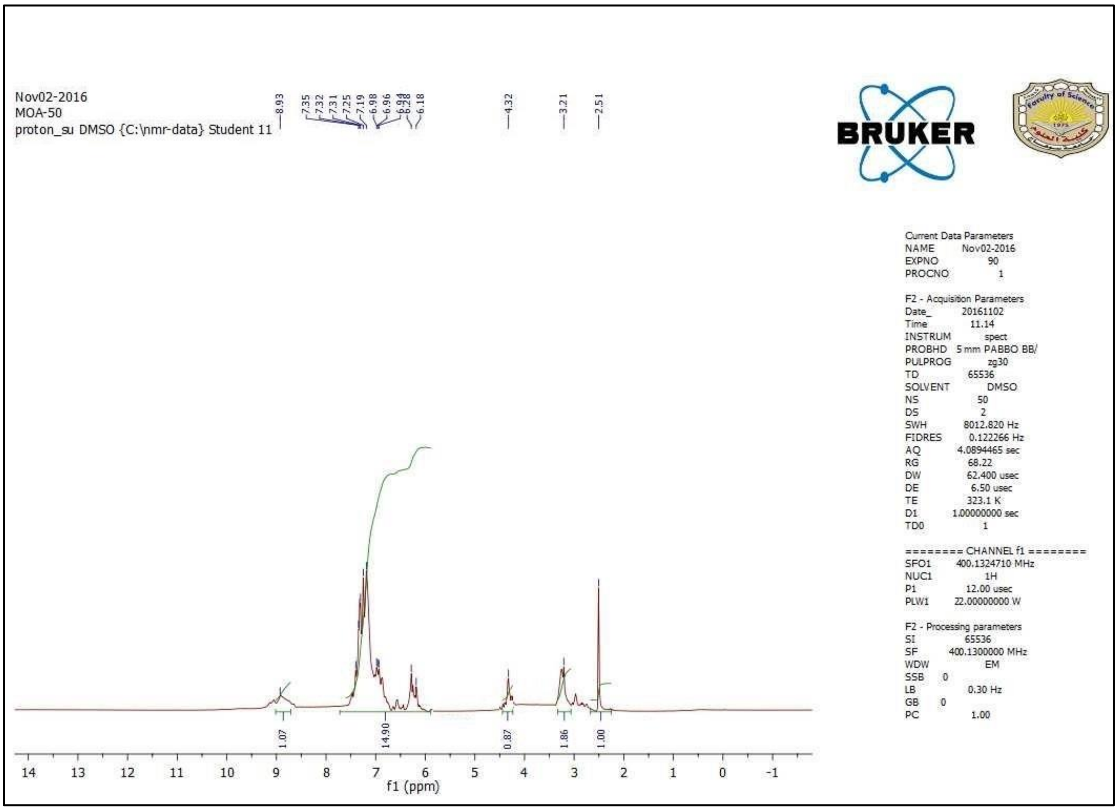

Figure S25. ^{1}H-NMR spectrum of compound 11

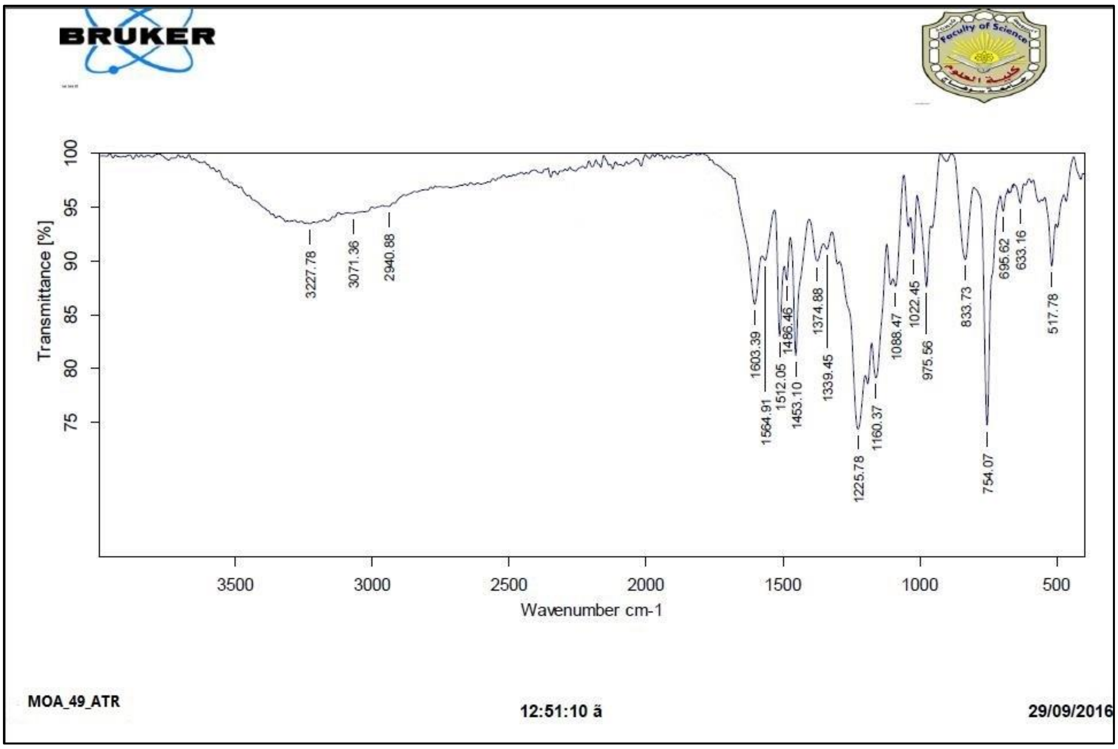

Figure S26. IR spectrum of compound 12

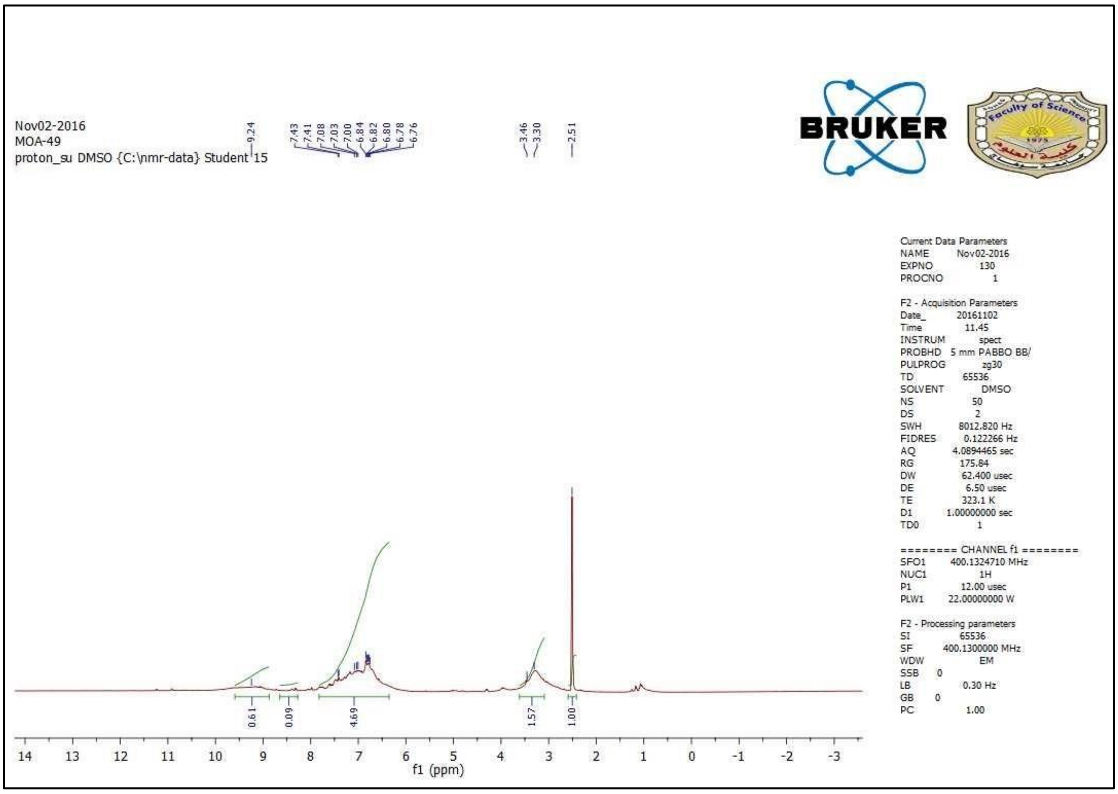

Figure S27. ^{1}H-NMR spectrum of compound 12

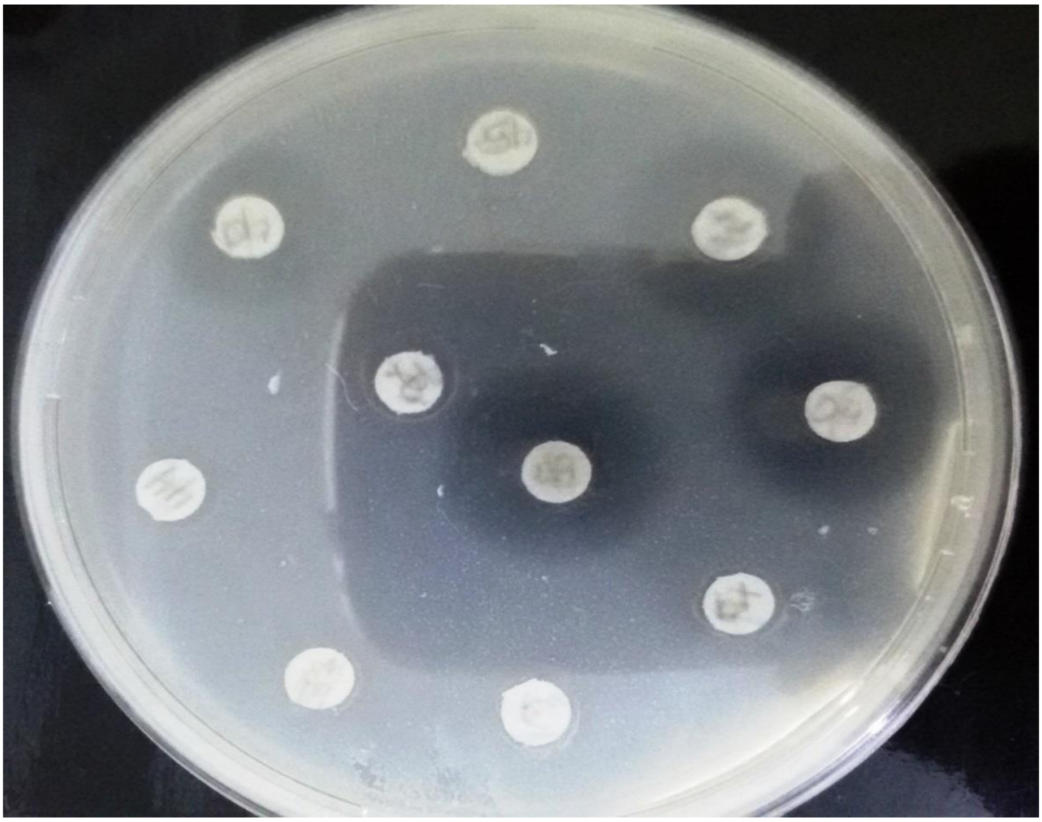

Figure S28. Antibacterial activity of the tested compounds.

Compound 5a

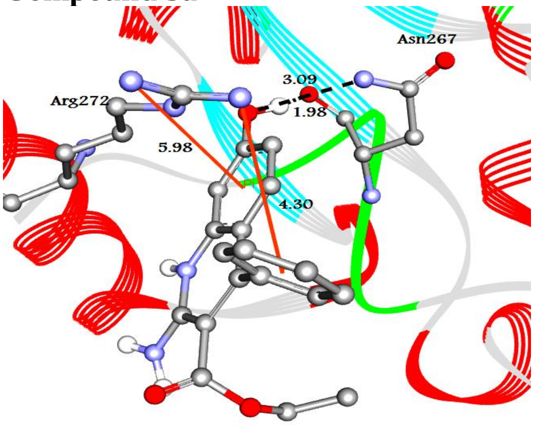

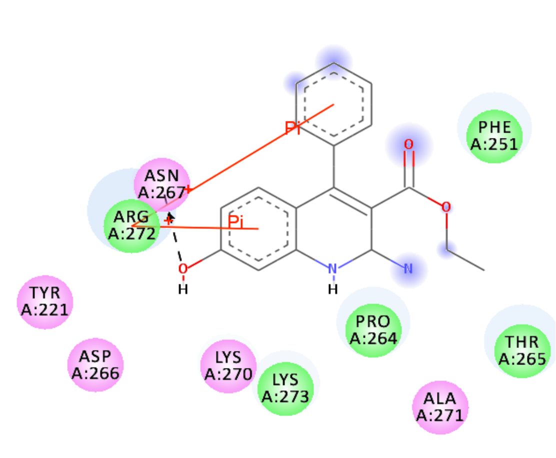

Compound 5b

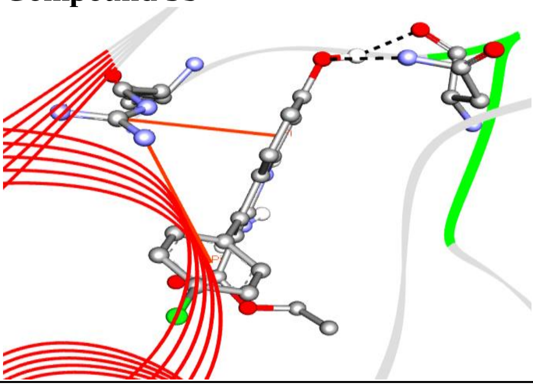

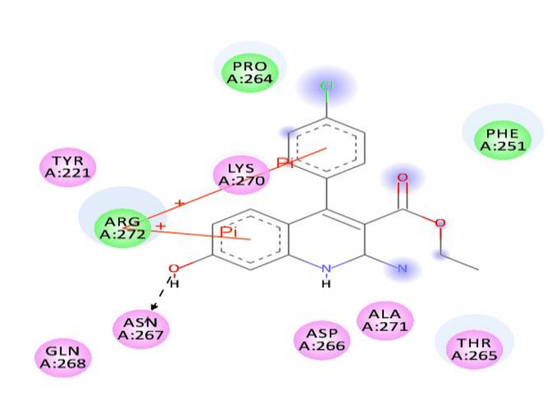

Compound 5c

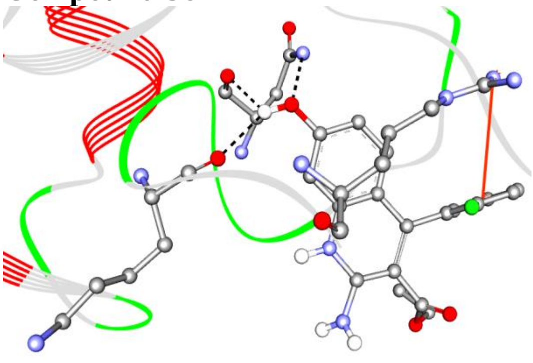

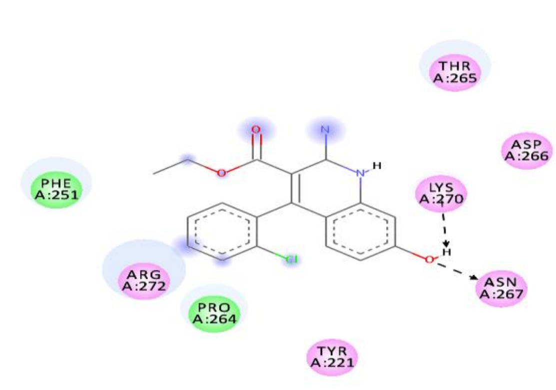

Compound 5d

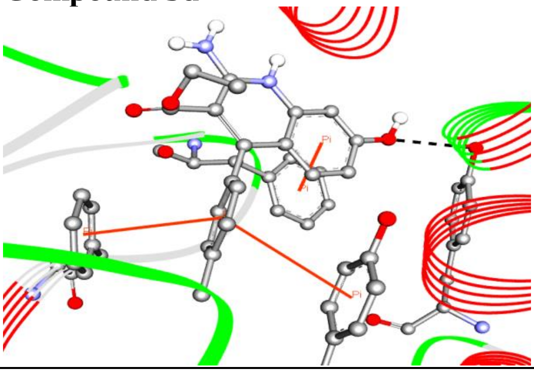

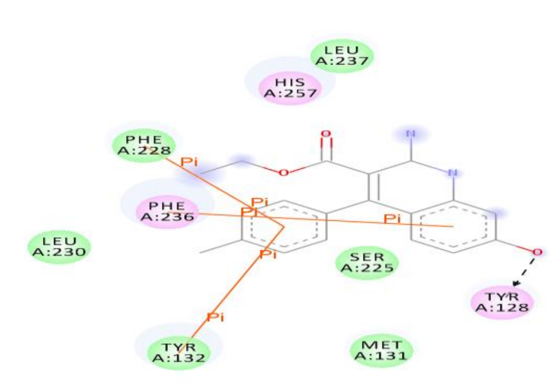

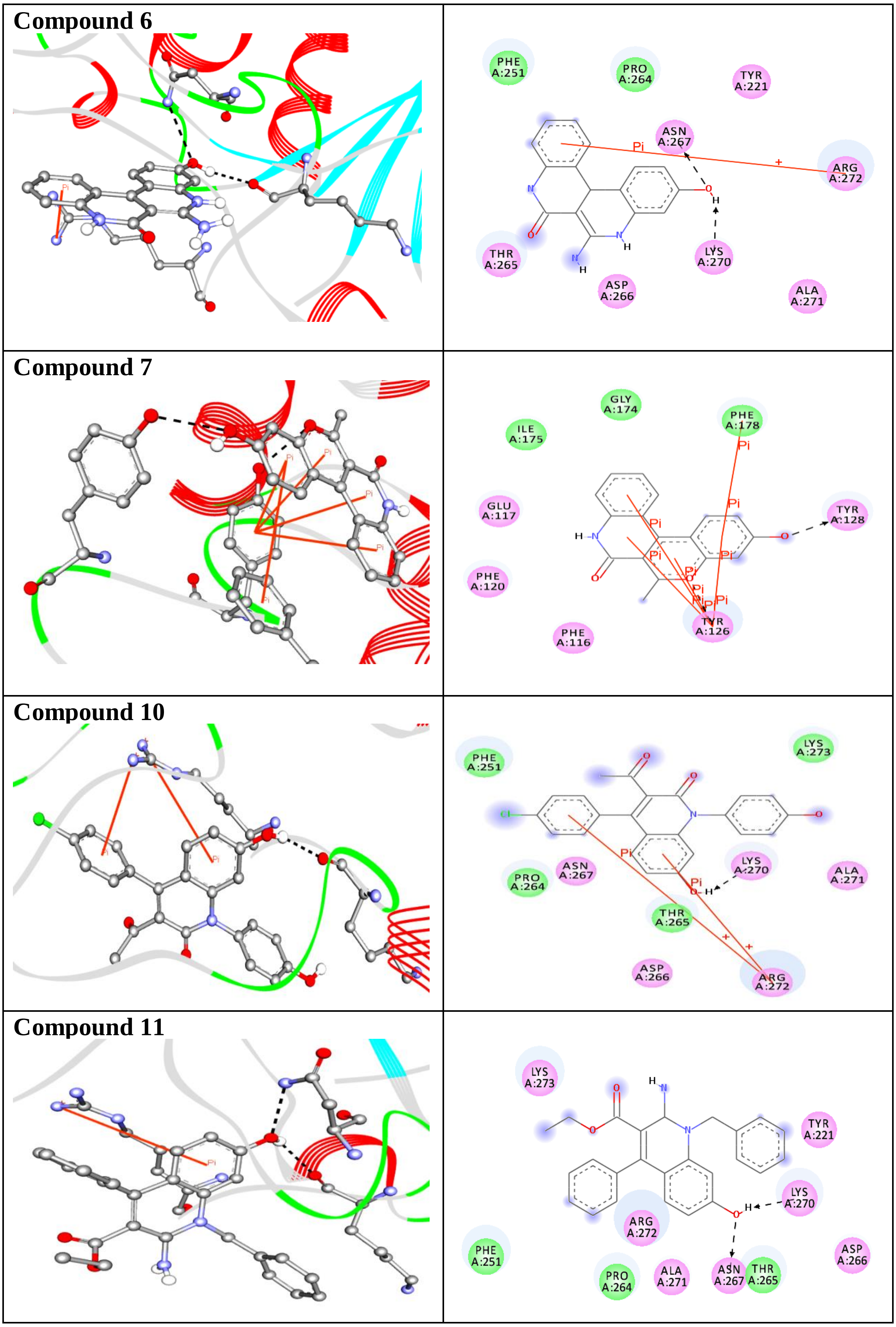

A B

Figure S29. (A) 3D, (B) 2D representations of the other docked compound with 1DXO. H-bonds are represented in black dotted lines while pi-interactions are shown in orange lines.

Compound 5a

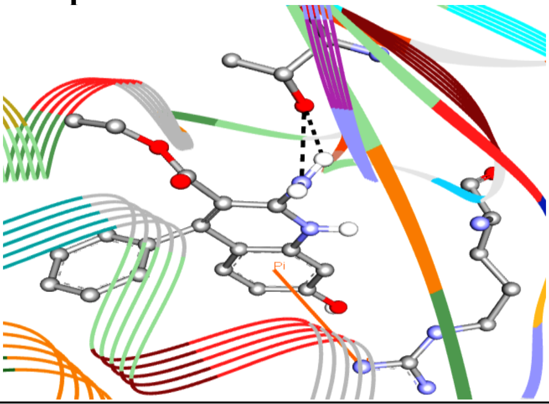

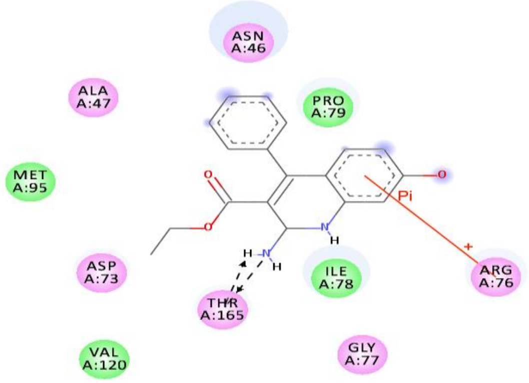

Compound 5b

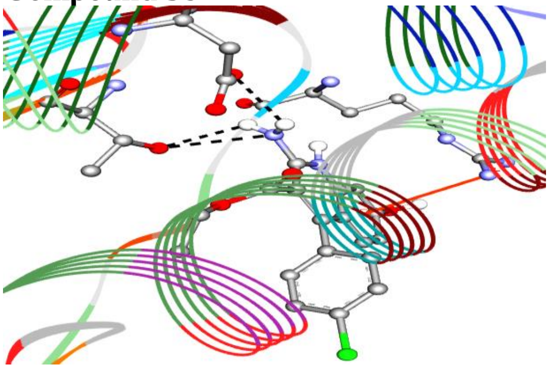

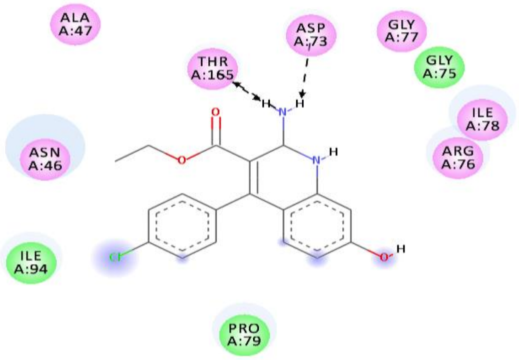

Compound 5c

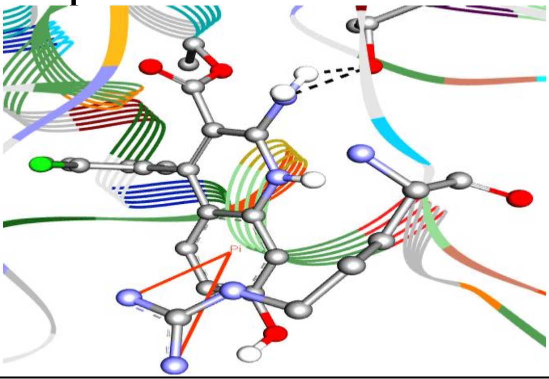

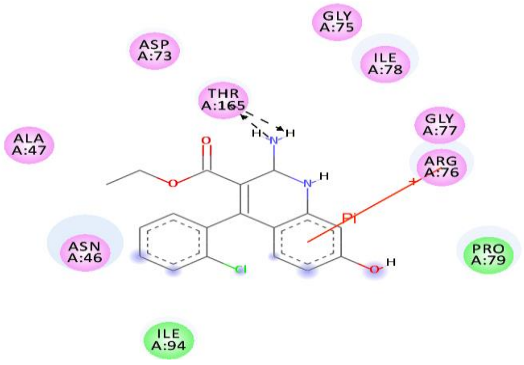

Compound 5d

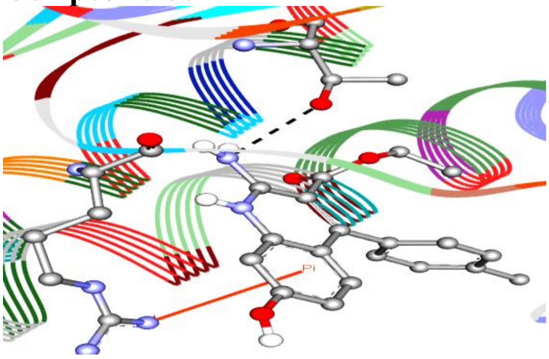

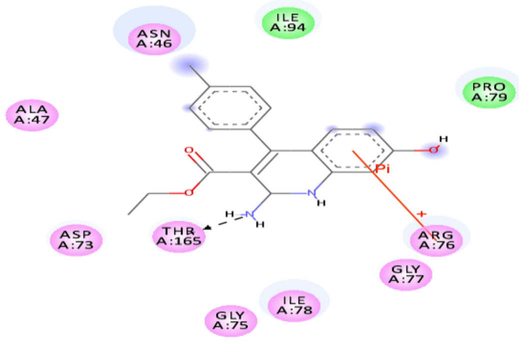

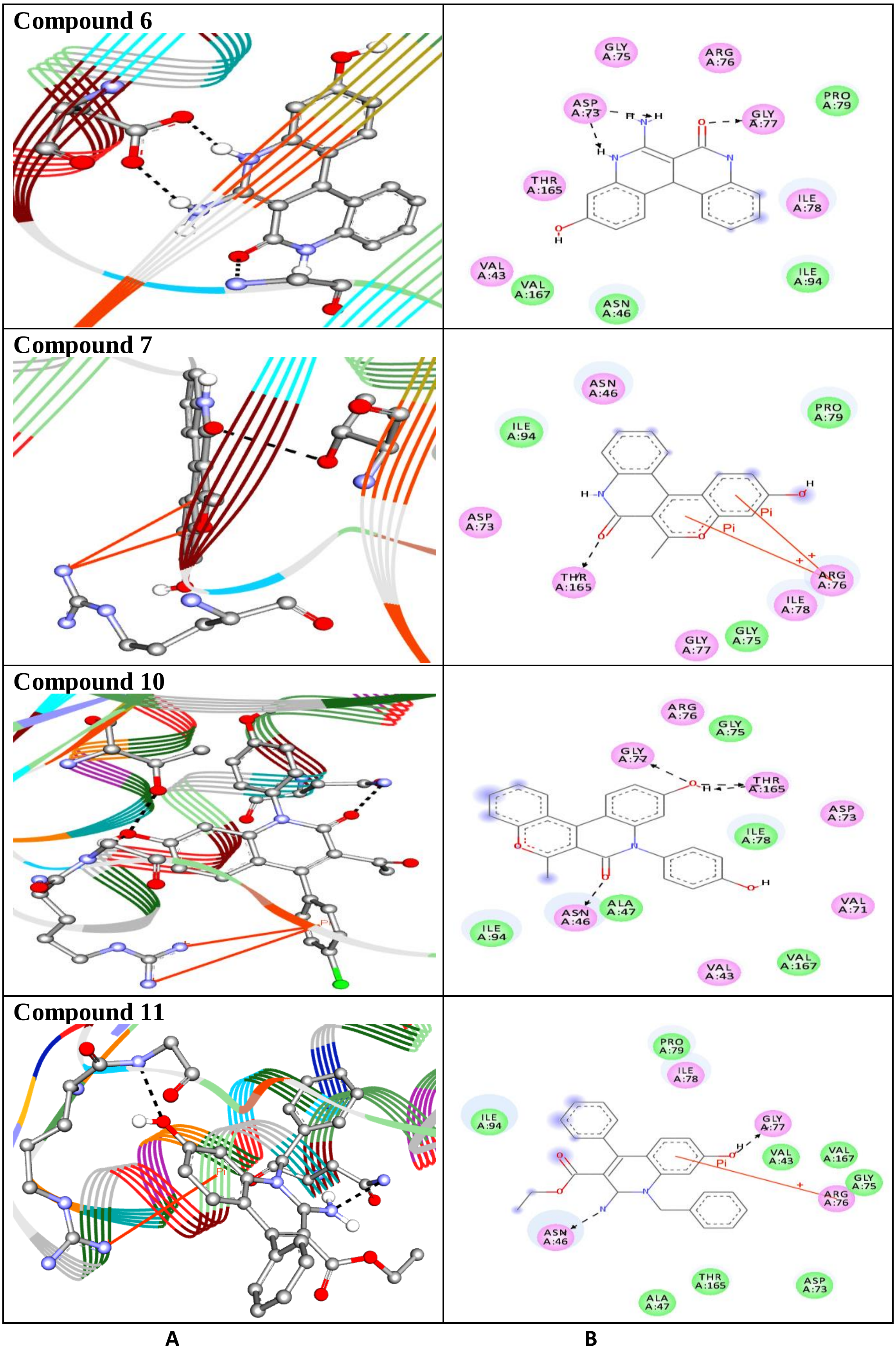

Figure S30. (A) 3D, (B) 2D representations of the other docked compound with 1JA6. H-bonds are represented in black dotted lines while pi-interactions are shown in orange lines.
